# The transcriptional regulator SsrB is involved in a molecular switch controlling virulence lifestyles of *Salmonella*

**DOI:** 10.1371/journal.ppat.1006497

**Published:** 2017-07-13

**Authors:** Deyanira Pérez-Morales, María M. Banda, N. Y Elizabeth Chau, Heladia Salgado, Irma Martínez-Flores, J. Antonio Ibarra, Bushra Ilyas, Brian K. Coombes, Víctor H. Bustamante

**Affiliations:** 1 Departamento de Microbiología Molecular, Instituto de Biotecnología, Universidad Nacional Autónoma de México, Cuernavaca, Morelos, México; 2 Department of Biochemistry and Biomedical Sciences, McMaster University, Hamilton, Ontario, Canada; 3 Michael G. DeGroote Institute for Infectious Disease Research, McMaster University, Hamilton, Ontario, Canada; 4 Programa de Genómica Computacional, Centro de Ciencias Genómicas, Universidad Nacional Autónoma de México, Cuernavaca, Morelos, México; 5 Departamento de Microbiología, Escuela Nacional de Ciencias Biológicas, Instituto Politécnico Nacional, Ciudad de México, México; Children's Hospital Boston, UNITED STATES

## Abstract

The evolution of bacterial pathogenicity, heavily influenced by horizontal gene transfer, provides new virulence factors and regulatory connections that alter bacterial phenotypes. *Salmonella* pathogenicity islands 1 and 2 (SPI-1 and SPI-2) are chromosomal regions that were acquired at different evolutionary times and are essential for *Salmonella* virulence. In the intestine of mammalian hosts, *Salmonella* expresses the SPI-1 genes that mediate its invasion to the gut epithelium. Once inside the cells, *Salmonella* down-regulates the SPI-1 genes and induces the expression of the SPI-2 genes, which favor its intracellular replication. The mechanism by which the invasion machinery is deactivated following successful invasion of host cells is not known. Here, we show that the SPI-2 encoded transcriptional regulator SsrB, which positively controls SPI-2, acts as a dual regulator that represses expression of SPI-1 during intracellular stages of infection. The mechanism of this SPI-1 repression by SsrB was direct and acts upon the *hilD* and *hilA* regulatory genes. The phenotypic effect of this molecular switch activity was a significant reduction in invasion ability of *S*. *enterica* serovar Typhimurium while promoting the expression of genes required for intracellular survival. During mouse infections, *Salmonella* mutants lacking SsrB had high levels of *hilA* (SPI-1) transcriptional activity whereas introducing a constitutively active SsrB led to significant *hilA* repression. Thus, our results reveal a novel SsrB-mediated mechanism of transcriptional crosstalk between SPI-1 and SPI-2 that helps *Salmonella* transition to the intracellular lifestyle.

## Author summary

*Salmonella* infect humans and a wide range of mammalian hosts. Successful infection requires the bacteria to sense their surroundings and regulate gene expression in a way that maximizes fitness in that particular environment. The two major lifestyles of *Salmonella* include extracellular stages and intracellular stages of host cell infection; however, the molecular mechanisms of how *Salmonella* transitions between these two lifestyles are not completely understood. Here we show that the transcriptional regulator SsrB functions in a dual capacity, activating genes required for intracellular survival while simultaneously repressing genes needed for extracellular stages of infection. Our data highlight how regulatory crosstalk is selective during infection, presumably because it helps facilitate rapid transitions in bacterial lifestyles that ultimately promote bacterial survival and replication.

## Introduction

All organisms carefully regulate gene expression to ensure correct spatiotemporal deployment of gene products. For bacterial pathogens that reside in multiple niches, a mechanism to coordinate gene expression with environmental sensing is crucial for their ability to cause disease. This is achieved largely by two-component regulatory systems that sense external surroundings using a membrane sensor kinase that signals to a cytosolic response regulator that directs a transcriptional response [1].

In *Salmonella*, many of virulence genes required for infection are found in horizontally acquired pathogenicity islands [2]. *Salmonella* pathogenicity islands 1 and 2 (SPI-1 and SPI-2) were acquired at different evolutionary times and have key roles in *Salmonella* virulence [3, 4]. Both SPI-1 and SPI-2 encode a type III secretion system (T3SS), effector proteins, chaperones, and transcriptional regulators that control the expression of the genes within each of the SPIs [3, 5]. The SPI-1-encoded T3SS (T3SS-1) and effector proteins mediate *Salmonella* invasion of host cells leading to gastroenteritis [3, 4]. Following invasion, the genes within SPI-2 are required for *Salmonella* survival and replication within its intracellular niche, the *Salmonella*-containing vacuole (SCV). The ability of *Salmonella* to replicate inside macrophages allows for dissemination, leading to systemic disease in susceptible hosts [3, 4].

Consistent with their function, the SPI-1 genes are expressed when *Salmonella* is in the intestinal lumen or associated with the epithelium [6]. SPI-1 is also expressed in a subpopulation of bacteria that replicates in the cytosol of cultured epithelial cells [7]. The SPI-2 genes are mainly expressed when *Salmonella* is inside the SCV of epithelial cells and macrophages [7–11]. *In vitro*, SPI-1 genes are expressed when *Salmonella* is grown to early stationary phase in nutrient-rich lysogeny broth (LB), whereas SPI-2 genes are expressed when *Salmonella* is grown to late stationary phase in LB or in acidic minimal media containing micromolar concentrations of phosphate and magnesium ions [12–14].

A transcriptional regulatory cascade comprised of HilD, HilA and InvF, positively controls the expression of the SPI-1 genes as well as several other genes outside this island that are required for *Salmonella* invasion of host cells [3, 15–17]. When *Salmonella* is grown to late stationary phase in LB, HilD mediates transition of the gene expression program from SPI-1 to SPI-2 through activation of the SsrA-SsrB two-component system, a master regulator of SPI-2 genes [14]. In response to chemical cues detected inside host cells, the SsrA sensor kinase (also called SpiR) phosphorylates the SsrB response regulator leading to the activation of the genes found within SPI-2 and in other regions of the genome [3, 18, 19]. SsrB binds to a degenerate A+T-rich 18-bp palindrome sequence [20], probably making few base contacts; however, the exact mechanism by which SsrB interacts with DNA may vary from gene to gene [21].

The mechanism by which the invasion machinery is repressed following invasion of host cells is not known. Here, we report that SsrB represses the expression of SPI-1 genes directly by acting on the *hilD* and *hilA* regulatory genes. Following invasion of macrophage cells SsrB represses expression of the invasion machinery encoded in the SPI-1 genes, while activating expression of the SPI-2 genes needed for intracellular survival. Consistent with this model, *Salmonella* mutants lacking SsrB had high levels of *hilA* transcriptional activity during mouse infections, whereas introducing a constitutively active SsrB led to significant *hilA* repression *in vivo*. Thus, our results reveal a regulatory switch activity for SsrB that helps *Salmonella* transition to the intracellular environment.

## Results

### SsrB represses the expression of SPI-1 genes

**Fig 1:**
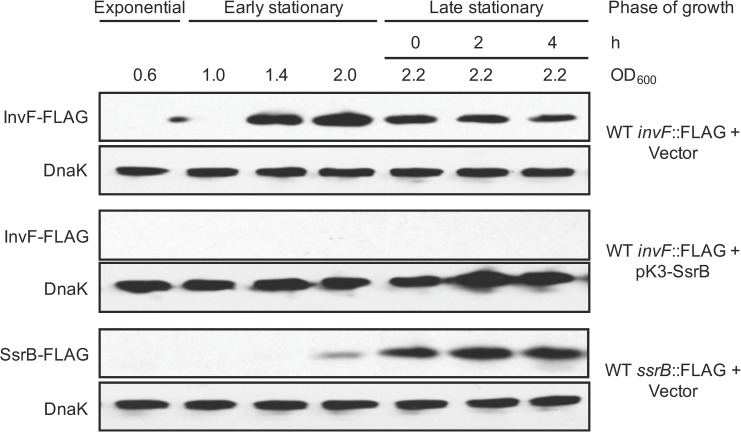
SsrB represses the expression of the SPI-1-encoded regulator InvF. Expression of InvF-FLAG and SsrB-FLAG in the WT *S*. Typhimurium strain containing the plasmid pK3-SsrB expressing SsrB from a constitutive promoter, or the vector pMPM-K3, was analyzed by Western blot using monoclonal anti-FLAG antibodies. Whole cell lysates were prepared from samples of bacterial cultures grown in LB at 37°C, at the OD_600_ or the time indicated, representing exponential, early stationary or late stationary phases of growth. As a loading control, the expression of DnaK was also determined using monoclonal anti-DnaK antibodies.

**Fig 2:**
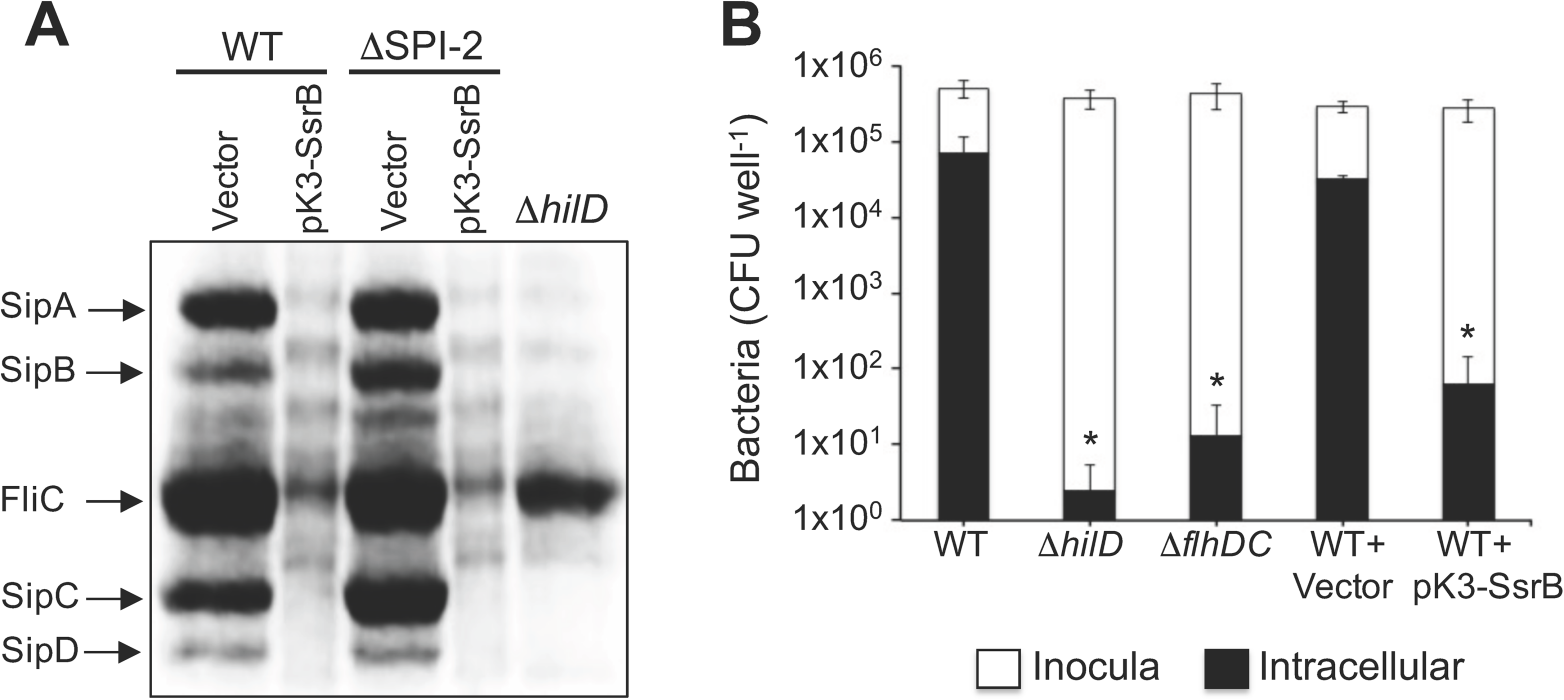
SsrB represses the secretion of SPI-1-encoded proteins and *Salmonella* invasion of HeLa cells. (A) Secretion profiles of the SPI-1-encoded proteins SipA, SipB, SipC and SipD were examined in the WT *S*. Typhimurium strain and its isogenic ΔSPI-2 mutant containing the plasmid pK3-SsrB that constitutively expresses SsrB, or the vector pMPM-K3, grown for 9 h in LB at 37°C. As a control, the secretion profile for the Δ*hilD* mutant that lacks the SipA-D proteins is also shown. FliC is the major subunit of the flagellar filament. (B) HeLa cells were infected with WT *S*. Typhimurium or isogenic Δ*hilD* and Δ*flhDC* mutants containing either pK3-SsrB or vector control pMPM-K3, and intracellular bacteria enumerated after 1 hr. White and black columns indicate the number of bacteria from the starting inoculum and from intracellular bacteria recovered from the HeLa cells, respectively. Data represents the mean with standard deviation of three independent experiments. *Statistically different values with respect to the WT strain with or without the vector pMPM-K3, *P* < 0.005.

In a previous study we showed that SPI-1 and SPI-2 genes are expressed during early and late stationary phase, respectively, when *S*. Typhimurium is grown in LB [14]. Interestingly, the expression of SsrB during late stationary phase coincided with down-regulation of the SPI-1 regulator HilA [14]. To investigate the mechanisms controlling this regulation, we examined the chromosomal expression of InvF-FLAG by Western blot in a wild-type (WT) *S*. Typhimurium strain that constitutively expresses SsrB from the pK3-SsrB plasmid, or a strain containing the vector control pMPM-K3. InvF is a SPI-1 regulator whose expression is dependent on HilA [3]. The chromosomal expression of SsrB-FLAG was also assessed as a control in the strain containing pMPM-K3. As expected, in the presence of the vector pMPM-K3 the protein level of InvF-FLAG was maximal in early stationary phase and decreased during late stationary phase, whereas expression of SsrB-FLAG was induced only during late stationary phase (Fig 1). In contrast, in the presence of the pK3-SsrB plasmid InvF-FLAG was not detected at any of the time points tested (Fig 1), indicating that SsrB expression leads to InvF repression. To examine the broader impact of SsrB on SPI-1, we determined the effect of SsrB on the effector secretion profile in WT *S*. Typhimurium grown in LB. Consistent with the results with InvF, in cells constitutively expressing SsrB there was reduced secretion of the SPI-1-encoded effectors SipA, SipB, SipC and SipD, as well as the flagellar protein FliC, in the culture supernatants (Fig 2A). Similar results were obtained using a *S*. Typhimurium ΔSPI-2 mutant (Fig 2A), indicating that the repressing effect of SsrB on the secretion of SipA-D and FliC proteins does not require any other SPI-2-encoded factor. Together, these results show that SsrB represses the expression of the SPI-1 and flagellar genes.

### Expression of SsrB decreases *S*. Typhimurium invasion of HeLa cells

Invasion of *Salmonella* into host cells requires the cellular functions encoded in both the SPI-1 and flagellar genes [3, 22, 23]. Thus, we used gentamicin protection assays to determine whether SsrB-mediated repression of the SPI-1 and flagellar genes had a phenotypic consequence on bacterial invasion. HeLa cells were infected with WT *S*. Typhimurium containing the pK3-SsrB plasmid or the pMPM-K3 vector and the number of intracellular bacteria was determined 1 h post-infection. *S*. Typhimurium Δ*hilD* and Δ*flhDC* mutants, lacking master positive regulators for the SPI-1 and flagellar genes, respectively, were used as controls. The constitutive expression of SsrB from pK3-SsrB resulted in a 500-fold reduction in invasion (Fig 2B). As expected, the Δ*hilD* and Δ*flhDC* mutants also showed a very strong reduction in invasion (Fig 2B). These results show that constitutive expression of SsrB negatively affects *Salmonella* invasion of HeLa cells, consistent with its ability to repress SPI-1 and flagellar genes.

### SsrB represses the SPI-1 regulatory cascade

**Fig 3:**
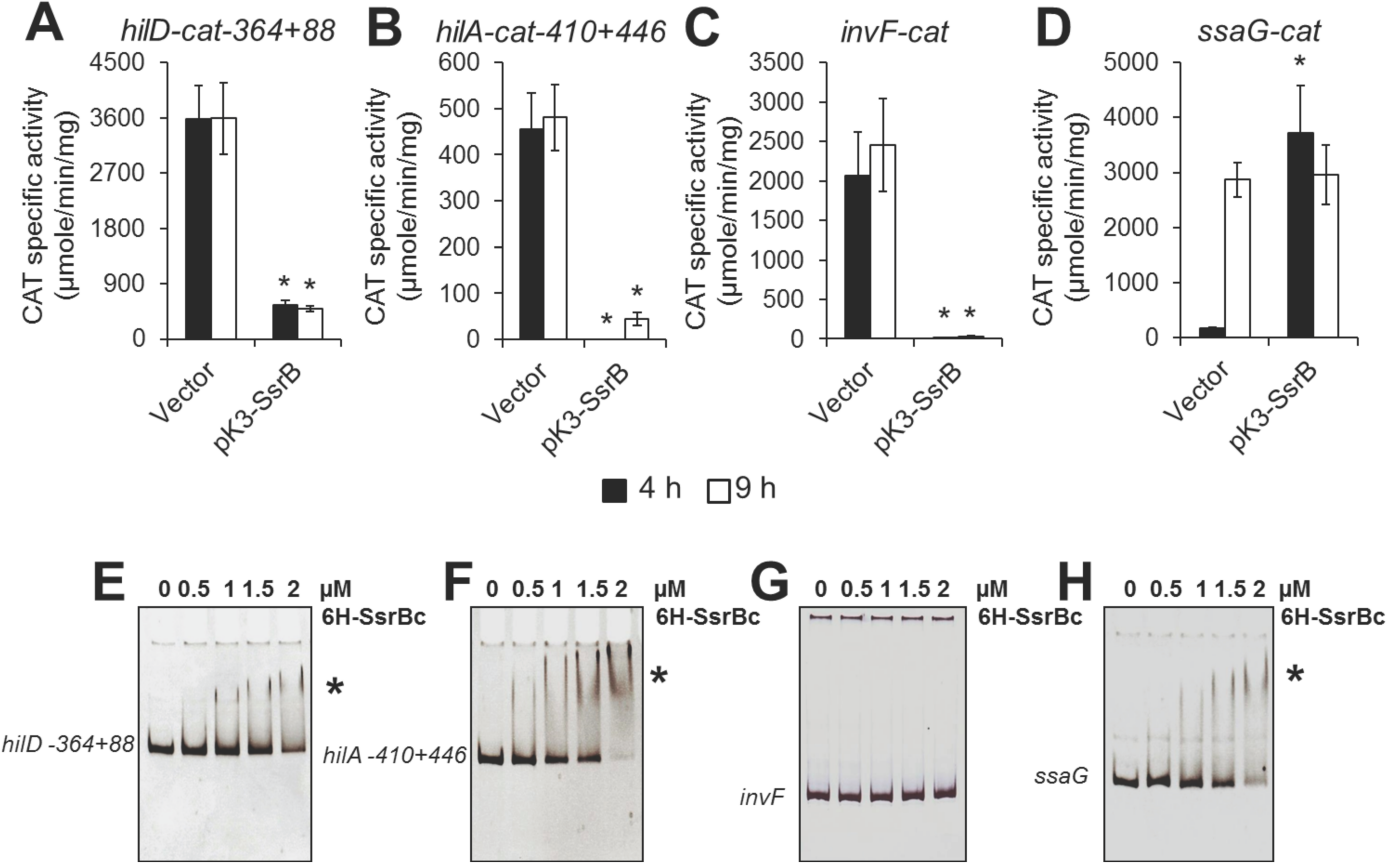
SsrB directly represses the *hilD* and *hilA* SPI-1 regulatory genes. Expression of the *hilD-cat-364+88* (A), *hilA-cat-410+446* (B), *invF-cat* (C) and *ssaG-cat* (D) transcriptional fusions was tested in the WT *S*. Typhimurium strain containing the vector pMPM-K3 or the plasmid, pK3-SsrB, which expresses SsrB from a constitutive promoter. The CAT-specific activity was determined from samples collected of bacterial cultures grown for 4 and 9 h in LB at 37°C. Data represents the mean with standard deviation of three independent experiments. *Statistically different values with respect to the WT strain containing the vector pMPM-K3, *P* < 0.0005. SsrB binding to the DNA fragments contained in the *hilD-cat-364+88* (E), *hilA-cat-410+446* (F), *invF-cat* (G) and *ssaG-cat* (H) fusions were analyzed using EMSAs. The respective PCR-amplified and purified DNA fragments were incubated with increasing concentrations of purified 6H-SsrBc (0, 0.5, 1, 1.5 and 2 μM). DNA-protein complexes are indicated by an asterisk.

The SPI-1-encoded regulators HilD, HilA and InvF positively control the expression of the genes within this island in a cascade fashion, where HilD induces the expression of HilA and it, in turn, activates the expression of InvF [3, 24]. To investigate how SsrB represses the SPI-1 genes, we analyzed the effect of constitutive SsrB expression on the transcription of *hilD*, *hilA* and *invF*, using *cat* transcriptional fusions. As controls for these assays the expression of *sirA* and *csrA*, which are found outside SPI-1 and encode known regulators of the SPI-1 genes, and *ssaG*, a SPI-2 gene whose expression is dependent on SsrB [3, 24], was also tested using *cat* transcriptional fusions. Constitutive expression of SsrB from pK3-SsrB nearly abolished the expression of the *hilD*-*cat-364+88*, *hilA*-*cat-410+446* and *invF*-*cat* fusions, in bacterial cultures grown in LB for 4 and 9 h, times representing early and late stationary phase of growth (Fig 3A, 3B and 3C). In contrast, SsrB had a non-significant effect on the expression of the *sirA-cat* and *csrA-cat* fusions (S1A and S1B Fig). SsrB induced the expression of the *ssaG-cat* fusion in the early stationary phase of growth, whereas in the presence of the pMPM-K3 vector its expression was only induced during late stationary phase (Fig 3D). This is consistent with previous results indicating that overexpression of SsrB can activate the SPI-2 genes even in the absence of its cognate sensor kinase SsrA, while still requiring its phosphorylable Asp56 residue [18]; since small inorganic phosphate donors, such as acetyl phosphate, can also phosphorylate SsrB [25]. Together, these results demonstrate that SsrB represses the transcription of the SPI-1 regulatory genes *hilD*, *hilA* and *invF*.

### SsrB directly represses *hilD* and *hilA*

To determine whether SsrB directly or indirectly represses the expression of *hilD*, *hilA*, and *invF*, we analyzed the interaction of SsrB with the regulatory regions of these genes by electrophoretic mobility shift assays (EMSAs). Full-length SsrB is unstable in solution, but the C-terminal DNA binding domain (6H-SsrBc) is stable and can specifically bind to promoter regions of SsrB-regulated genes [18, 25]. Therefore, purified 6H-SsrBc and the DNA fragments of each gene contained in the *hilD-*, *hilA-* and *invF-cat* fusions were used in these assays. 6H-SsrBc bound to the DNA fragments of *hilD* and *hilA* (Fig 3E and 3F) but did not bind to the DNA fragment of *invF* (Fig 3G). As expected, 6H-SsrBc also shifted the DNA fragment of *ssaG*, which was used as a positive control (Fig 3H) but it did not shift those of the *sirA* or *csrA* negative controls (S1C and S1D Fig). These results show that SsrB specifically binds to the regulatory regions of *hilD* and *hilA*.

**Fig 4:**
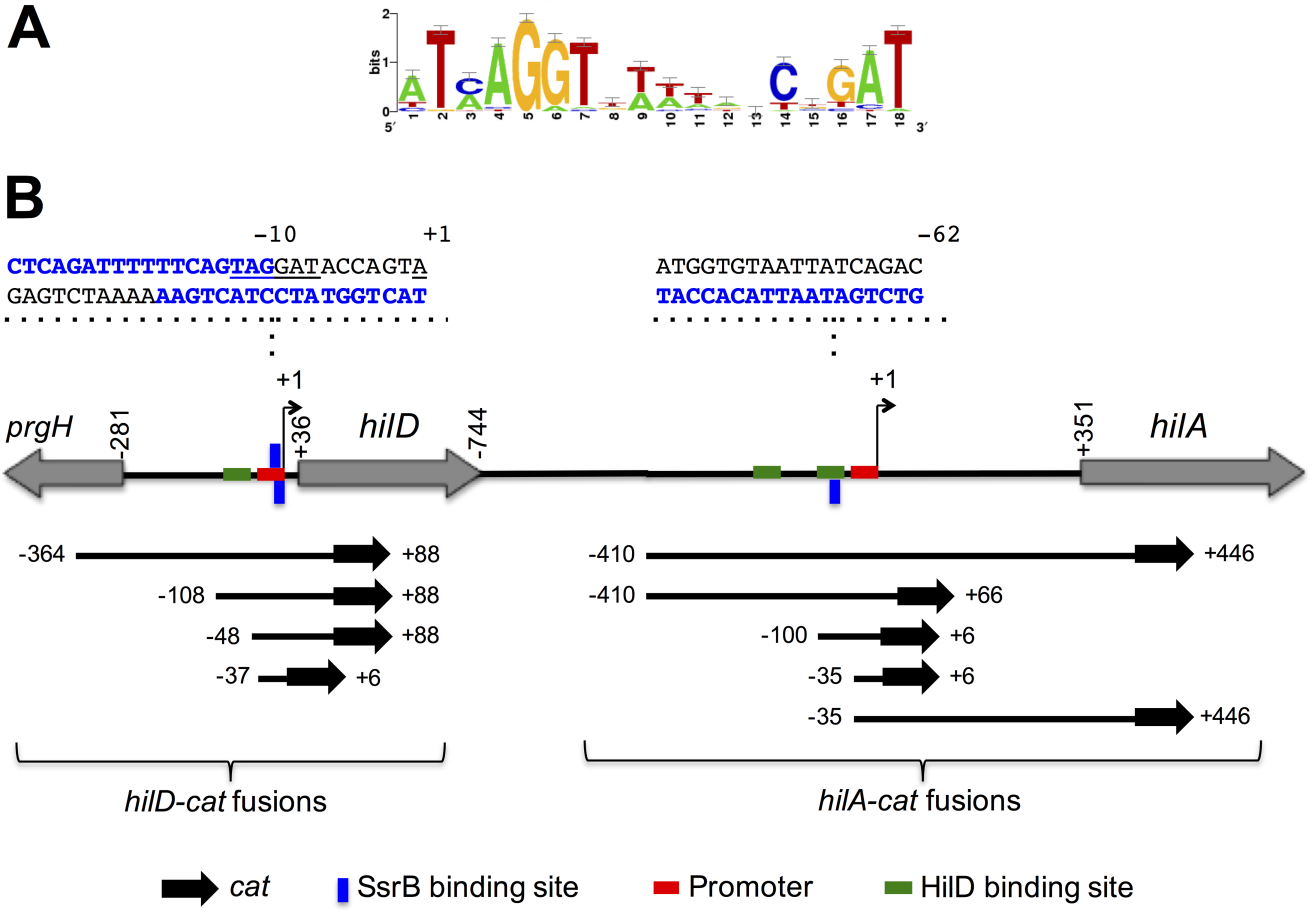
Schematic representation of the *hilD* and *hilA* genes and their regulatory elements. (A) Sequence logo for the PSSM used to predict the SsrB binding sites. (B) The locus containing *hilD* and *hilA*. The transcriptional start site (+1) of *hilD* and *hilA* is indicated by a bent arrow and red boxes represent their promoters. The SsrB-binding sites involved in repression of *hilD* or *hilA* are displayed as blue boxes below or above the respective regulatory region, which indicates the sense and anti-sense strand of DNA, respectively; their respective 18-bp sequence is shown. The HilD-binding sites on *hilD* and *hilA* are displayed as green boxes. The different *hilD-cat* and *hilA-cat* transcriptional fusions assessed in this study are also shown. All of the positions indicated are relative to the transcriptional start site of *hilD* or *hilA*.

Previous work has identified a conserved yet flexible 18 bp palindrome sequence that defines the SsrB binding sequence based on a position-specific scoring matrix [20]. Scanning with this sequence (Fig 4A) identified two putative SsrB-binding sites in the regulatory region of *hilD* and nine within the *hilA* regulatory region. Interestingly, the two putative SsrB-binding sites near *hilD* are located in the promoter, whereas in *hilA* one putative SsrB-binding site is located upstream of the promoter, overlapping a HilD-binding site, and the others are located far upstream or downstream of the promoter (Fig 4B).

**Fig 5:**
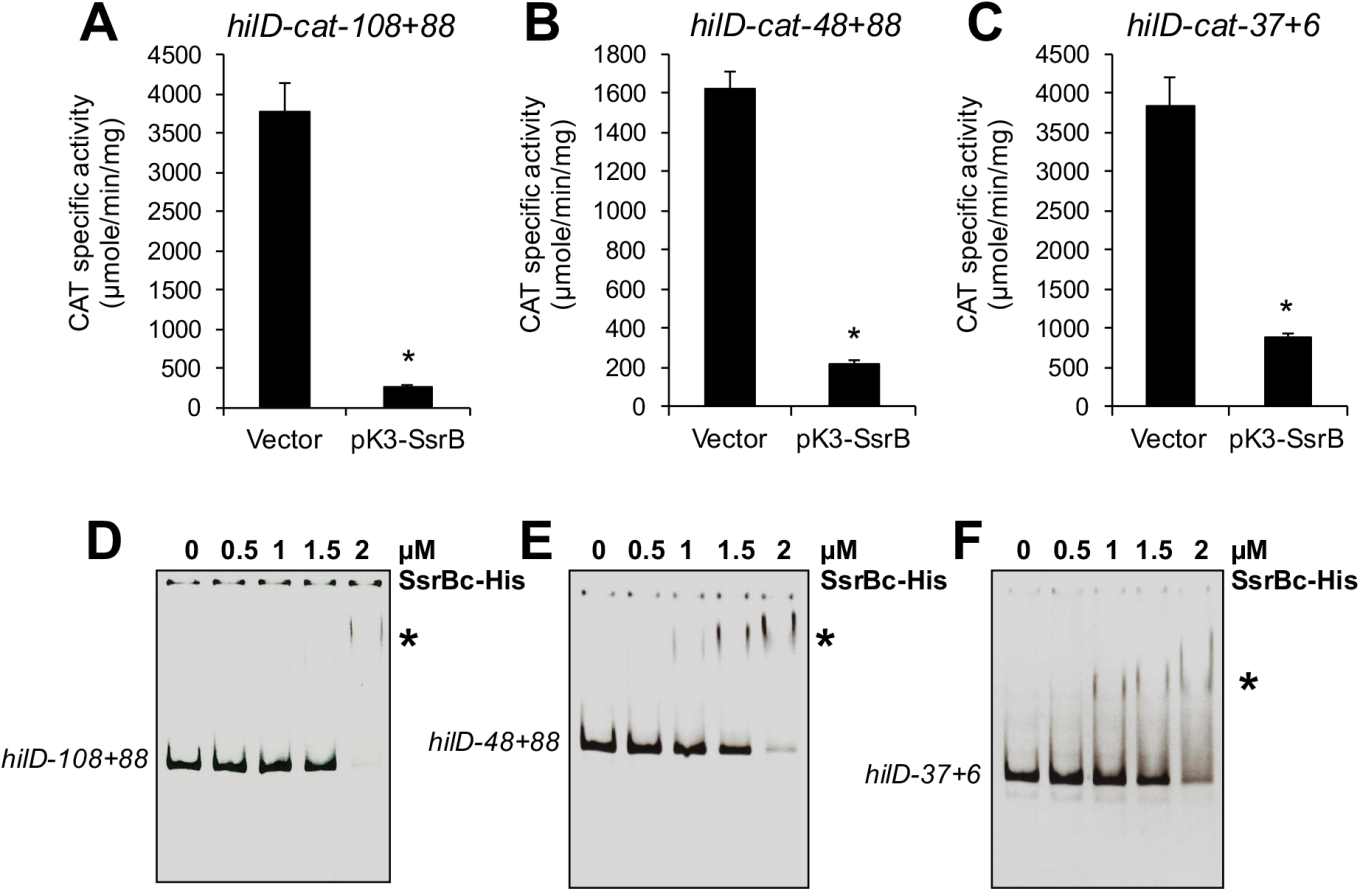
SsrB represses *hilD* by directly acting on its promoter. Expression of the *hilD-cat-108+88* (A), *hilD-cat-48+88* (B) and *hilD-cat-37+6* (C) transcriptional fusions was tested in the WT *S*. Typhimurium strain with the vector pMPM-K3, or the plasmid pK3-SsrB, which expresses SsrB from a constitutive promoter. The CAT-specific activity was determined from bacterial cultures grown for 9 h in LB at 37°C. Data represents the mean with standard deviation of three independent experiments. *Statistically different values with respect to the WT strain with pMPM-K3, *P* < 0.0005. EMSAs were performed to analyze whether SsrB binds to the *hilD* DNA fragments in the *hilD-cat-108+88* (D), *hilD-cat-48+88* (E) and *hilD-cat-37+6* (F) fusions. The DNA fragments were incubated with increasing concentrations of purified 6H-SsrBc (0, 0.5, 1, 1.5 and 2 μM). DNA-protein complexes are indicated by an asterisk.

To determine whether SsrB represses *hilD* through these two putative SsrB-binding sites, three different *cat* transcriptional fusions were constructed, each with distinct 5’ and 3’ deletions of the *hilD-cat-364+88* fusion that showed repression by SsrB (Fig 4B). The fusions (named according to the 5’ and 3’ positions of the *hilD* DNA fragment with respect to its transcriptional start site) *hilD-cat-108+88*, *hilD-cat-48+88* and *hilD-cat-37+6* were tested for CAT-specific activity in the presence of pK3-SsrB or the vector pMPM-K3. Positive autoregulation of *hilD* is not essential for its expression [26], therefore, the *hilD-cat-48+88* and *hilD-cat-37+6* fusions that lack the HilD-binding site upstream of *hilD*, were expected to be expressed. In the presence of pMPM-K3, *hilD-cat-108+88* reported expression levels similar to those from *hilD-cat-364+88* (compare Figs 5A and 3A), indicating that the *cis*-acting elements required for maximal expression of *hilD* are located between positions -108 to +88. In contrast, the expression of *hilD-cat-48+88* decreased by 50% relative to *hilD-cat-108+88* (Fig 5A and 5B), which is consistent with the reduction in *hilD* expression seen in the absence of autoregulation [26]. Interestingly, the *hilD-cat-37+6* fusion that contains only the promoter of *hilD* was activated to similar levels as the *hilD-cat-108+88* fusion (Fig 5A and 5C), demonstrating that in the absence of negative regulatory sequences between positions +6 to +88, the autoregulation is not required for maximal expression of *hilD*. Notably, the presence of pK3-SsrB significantly reduced the expression of *hilD-cat-108+88*, *hilD-cat-48+88* and *hilD-cat-37+6* (Fig 5A, 5B and 5C), indicating that SsrB negatively acts on the *hilD* promoter. EMSAs were performed to confirm that SsrB directly regulates the promoter of *hilD*. The *hilD* DNA fragments contained in *hilD-cat-108+88*, *hilD-cat-48+88* and *hilD-cat-37+6*, shifted in the presence of increasing concentrations of 6H-SsrBc (Fig 5D, 5E and 5F), indicating that SsrB binds to the promoter located between position -37 to +6 relative to the transcriptional start site of *hilD*, which is consistent with our bioinformatics analysis revealing two putative SsrB-binding sites on this region (Fig 4B). These results show that SsrB binds to the promoter of *hilD* and thus would repress its transcription.

**Fig 6:**
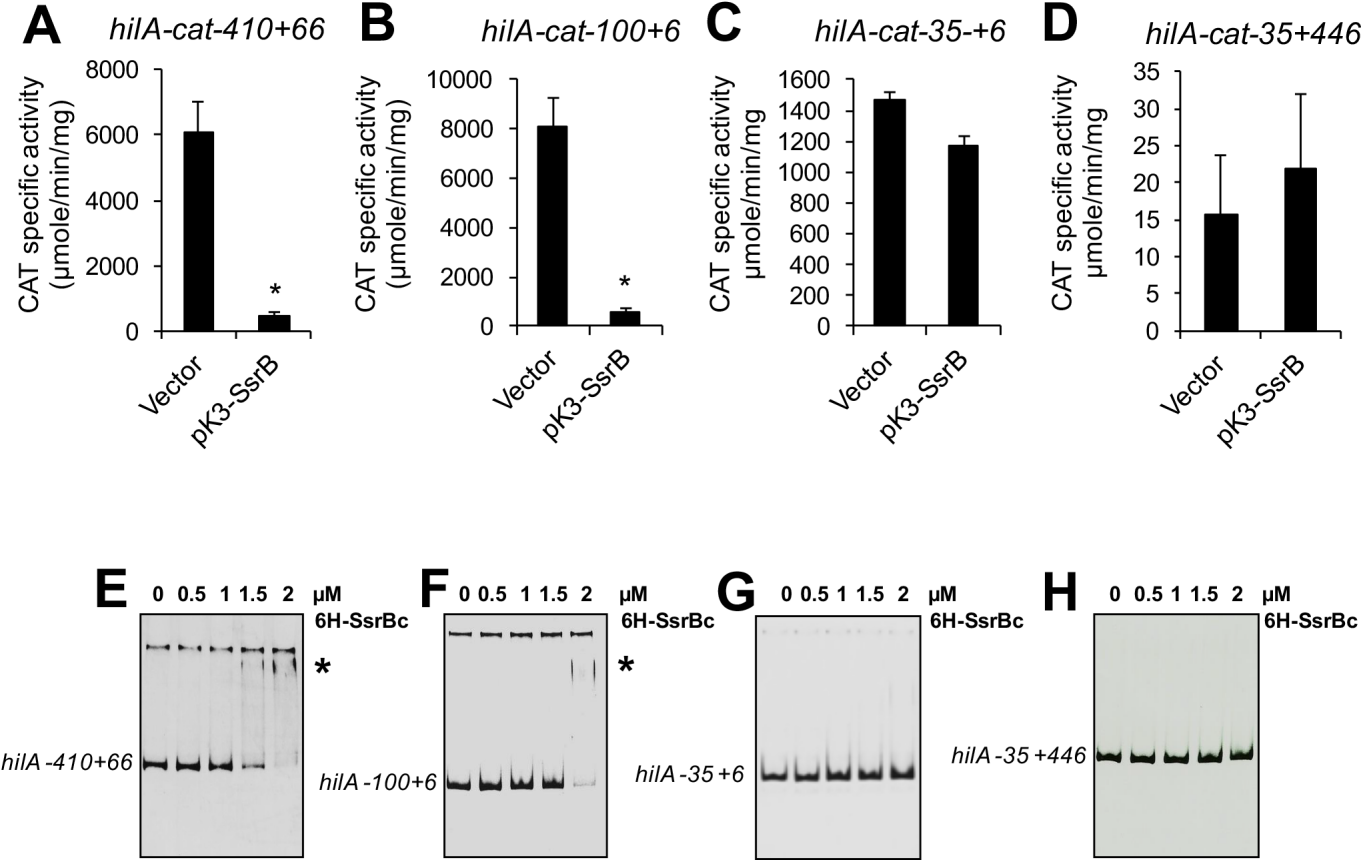
SsrB represses *hilA* by binding to the regulatory region between positions -100 to -35. Expression of the *hilA-cat-410+66* (A), *hilA-cat-100+6* (B), *hilA-cat-35+6* (C) and *hilA-cat-35+446* (D) transcriptional fusions was tested in the WT *S*. Typhimurium strain with the vector pMPM-K3, or the plasmid pK3-SsrB, which expresses SsrB from a constitutive promoter. The CAT-specific activity was determined from bacterial cultures grown for 9 h in LB at 37°C. Data represents the mean with standard deviation of three independent experiments. *Statistically different values with respect to the WT strain with pMPM-K3, *P* < 0.005. EMSAs were performed to determine whether SsrB binds to the *hilA* DNA fragments in the *hilA-cat-410+66* (E), *hilA-cat-100+6* (F), *hilA-cat-35+6* (G) and *hilA-cat-35+446* (H) fusions. The DNA fragments were incubated with increasing concentrations of purified 6H-SsrBc (0, 0.5, 1, 1.5 and 2 μM). DNA-protein complexes are indicated by an asterisk.

To determine whether SsrB mediates repression of *hilA* at any of the SsrB-binding sites we predicted bioinformatically, four different *hilA-cat* transcriptional fusions were constructed that have 5’ or 3’ deletions (or both) with respect to the *hilA-cat-410+446* fusion that showed repression by SsrB (Fig 4B). The fusions (named according to the 5’ and 3’ positions of the *hilA* DNA fragment with respect to its transcriptional start site) *hilA-cat-410+66*, *hilA-cat-100+6*, *hilA-cat-35+6* and *hilA-cat-35+446* were tested for CAT-specific activity in the presence of pK3-SsrB or the pMPM-K3 vector. Previously, it was shown that sequences flanking the promoter repress *hilA* and in the absence of the sequence upstream or downstream of the promoter, *hilA* was expressed independently of HilD [27–29]. Therefore, *hilA-cat-410+66*, *hilA-cat-100+6*, *hilA-cat-35+6* and *hilA-cat-35+446*, which lack the repressing sequences, were expected to be expressed at high levels, regardless of whether they contain the HilD binding sites or not. As expected, in the presence of the pMPM-K3 vector, *hilA-cat-410+66*, *hilA-cat-100+6* and *hilA-cat-35+6* were expressed at higher levels than *hilA-cat-410+446* (Fig 6A, 6B and 6C and Fig 3B). In contrast, the *hilA-cat-35+446* fusion, which lacks the sequence upstream of the promoter including the HilD-binding sites had severely reduced activity (Fig 6D). This suggests that the expression of *hilA* in the presence of the sequence downstream of the promoter, up to position +446, requires HilD. Notably, the presence of pK3-SsrB reduced the expression of *hilA-cat-410+66* and *hilA-cat-100+6*, but it did not affect the activity of *hilA-cat-35+6* and *hilA-cat-35+446* (Fig 6A, 6B, 6C and 6D), suggesting that SsrB mediates repression of *hilA* by acting on the region between -100 to -35. Expression analysis of *hilA-lux-740+35* and *hilA-lux-36+446* transcriptional fusions further indicated that this -100 to -35 region is needed for the SsrB-mediated repression of *hilA* (S2A and S2B Fig).

**Fig 7:**
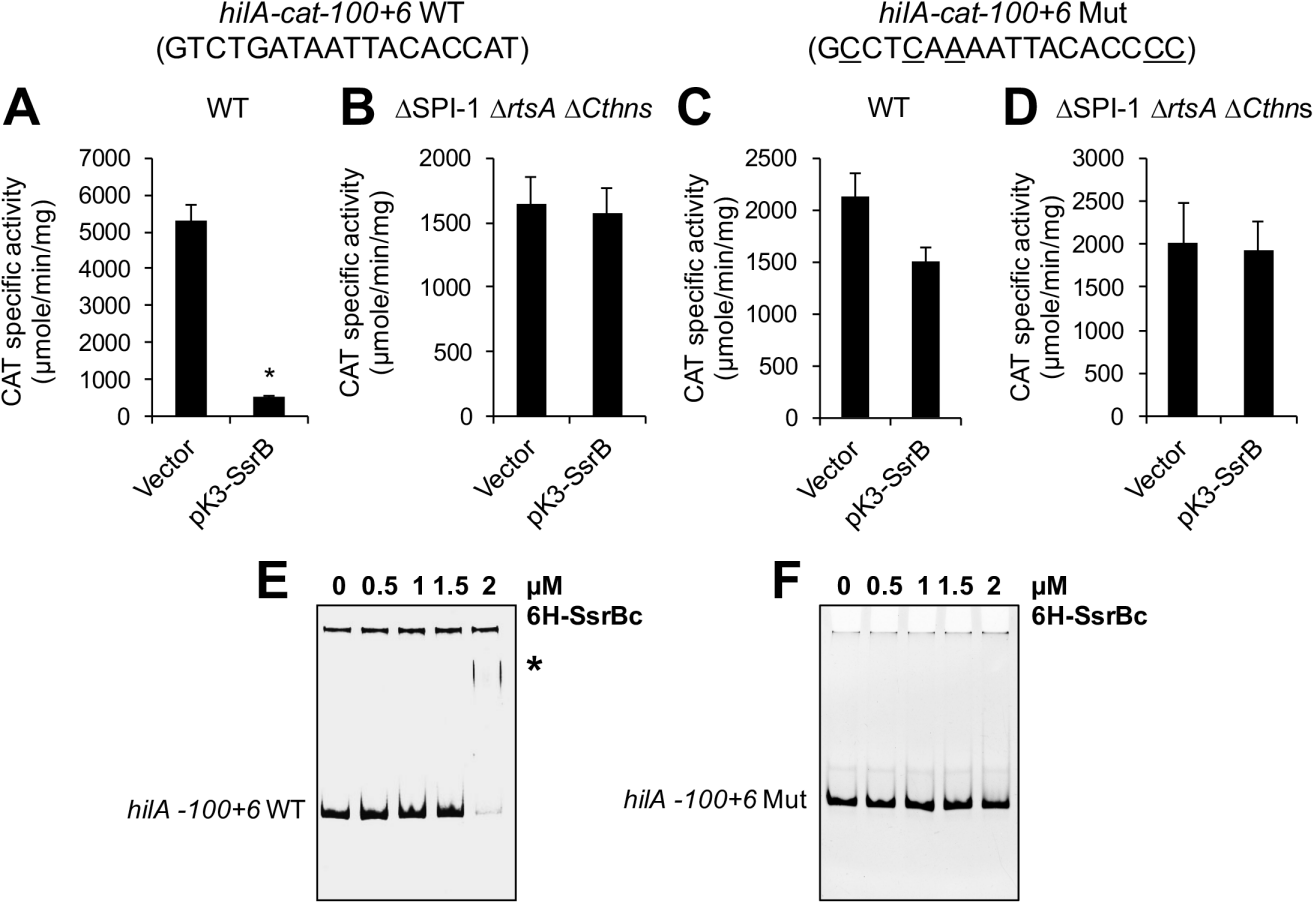
SsrB represses HilD-mediated expression of *hilA* by binding to a sequence overlapping the HilD-binding sequence upstream of the *hilA* promoter. Expression of the *hilA-cat-100+6* WT (wt SsrB binding site) (A and B) and *hilA-cat-100+6* Mut (mutated SsrB binding site) (C and D) fusions was determined in the WT *S*. Typhimurium strain (A and B) and its isogenic ΔSPI-1 Δ*rtsA* Δ*Cthns* mutant (C and D) that lacks HilD, HilC, RtsA and other regulators encoded in SPI-1, as well as H-NS. The CAT-specific activity was determined from bacterial cultures grown for 9 h in LB at 37°C. Data represents the mean with standard deviation of three independent experiments. *Statistically different values relative to the WT strain containing the pMPM-K3 vector, *P* < 0.0005. The WT and mutated SsrB-binding sequence are indicated; the nucleotides that were changed in the mutated sequence are underlined. EMSAs were performed to analyze the interaction of SsrB with the *hilA* DNA fragments carried by the *hilA-cat-100+6* WT (E) and *hilA-cat-100+6* Mut (F) fusions. The DNA fragments were incubated with increasing concentrations of purified 6H-SsrBc (0, 0.5, 1, 1.5 and 2 μM). DNA-protein complexes are indicated by an asterisk.

To determine whether SsrB physically interacts with this region of *hilA* we used EMSAs with purified 6H-SsrBc. 6H-SsrBc shifted the *hilA* DNA fragments contained in *hilA-cat-410+66* and *hilA-cat-100+6*, but not those contained in *hilA-cat-35+6* and *hilA-cat-35+446* (Fig 6E, 6F, 6G and 6H), indicating that SsrB binds between positions -100 to -35. These results are consistent with our bioinformatics analysis that predicted a SsrB-binding site in this region, centered at position -70, overlapping a HilD-binding site (Fig 4B). To determine whether SsrB mediates direct repression of *hilA* at this site, we mutated this site in the *hilA-cat-100+6* fusion by substituting five nucleotides within the predicted SsrB-binding site (Fig 7). The expression of the WT *hilA-cat-100+6* and mutated *hilA-cat-100+6* fusions was tested in WT *S*. Typhimurium containing pK3-SsrB or the vector control pMPM-K3. Constitutive expression of SsrB from pK3-SsrB drastically reduced the expression of the WT *hilA-cat-100+6* reporter but only slightly affected the activity of the mutated *hilA-cat-100+6* fusion (Fig 7A and 7C). Moreover, EMSAs showed that 6H-SsrBc binds to the *hilA* DNA fragment contained in WT *hilA-cat-100+6*, but does not bind to the *hilA-cat-100+6* fragment containing the mutated SsrB-binding site (Fig 7E and 7F). Interestingly, the mutations we created within the *hilA-cat-100+6* fusion also affected the regulation and binding of HilD on *hilA* (S3A, S3B, S3C and S3D Fig). These results show that SsrB represses *hilA* by binding to the site centered at position -70 that overlaps a HilD-binding site, which suggested that SsrB inhibits the HilD-mediated expression of *hilA*. To test this, the expression of the WT *hilA-cat-100+6* and mutated *hilA-cat-100+6* fusions was tested in a *S*. Typhimurium ΔSPI-1 Δ*rtsA* Δ*Cthns* triple mutant containing pK3-SsrB or the vector pMPM-K3. This mutant lacks HilD, HilC, RtsA and the other transcriptional regulators encoded in SPI-1, as well as the C-terminal region of H-NS. HilD, HilC and RtsA constitute a positive feed forward regulatory loop and each one can directly induce the expression of *hilA* [30]; on the other hand, in the absence of the C-terminal region of H-NS the expression of *hilA* is independent of HilD [26]. The presence of pK3-SsrB did not affect the HilD-, HilC- and RtsA-independent expression shown by the WT *hilA-cat-100+6* and mutated *hilA-cat-100+6* fusions in the ΔSPI-1 Δ*rtsA* Δ*Cthns* mutant (Fig 7B and 7D), which further indicates that SsrB inhibits the HilD-mediated expression of *hilA*. Taken together, these results strongly support that SsrB represses the expression of *hilA* by preventing HilD from binding. SsrB can also repress *hilA* through an indirect mechanism by negatively regulating the expression of *hilD*.

Notably, the *hilD* and *hilA* promoter sequences contained in the *hilD-cat-37+6* (directly repressed by SsrB) and *hilA-cat-35+6* (not repressed by SsrB) fusions, respectively, are 65% identical (S4 Fig); thus, only 15 different positions between these sequences determine binding and thus negative regulation of SsrB on the *hilD* promoter, but not on the *hilA* promoter.

### SsrB simultaneously represses SPI-1 and activates SPI-2 inside RAW264.7 mouse macrophages

Our results described above indicate that SsrB represses the expression of SPI-1 genes while activating expression of SPI-2 genes. In different *in vitro* SPI-2-inducing growth conditions that we have tested, *invF* was not de-repressed in the absence of SsrB (S5A and S5B Fig), consistent with the results from a previous study [17]. Thus, detection of specific environmental cues could be required for the repression of SPI-1 by SsrB in physiological conditions, which could occur during *Salmonella* infection of hosts. SPI-1 and SPI-2 are known to be inversely regulated when *Salmonella* is within macrophages [9–11, 31], an environment where SsrB is active [3]. To explore whether SsrB is involved in this inverse regulation during intracellular stages of infection, we analyzed the expression of *invF* (SPI-1) and *ssaG* (SPI-2) in WT bacteria and in bacteria lacking SsrB following macrophage infection. For this, transcriptional fusions of *invF* (SPI-1) and *ssaG* (SPI-2) to the luciferase operon (*lux*) were constructed in the pCS26-*Pac* vector. A *lux* transcriptional fusion of *hns*, a gene constitutively expressed, was also constructed as a control.

**Fig 8:**
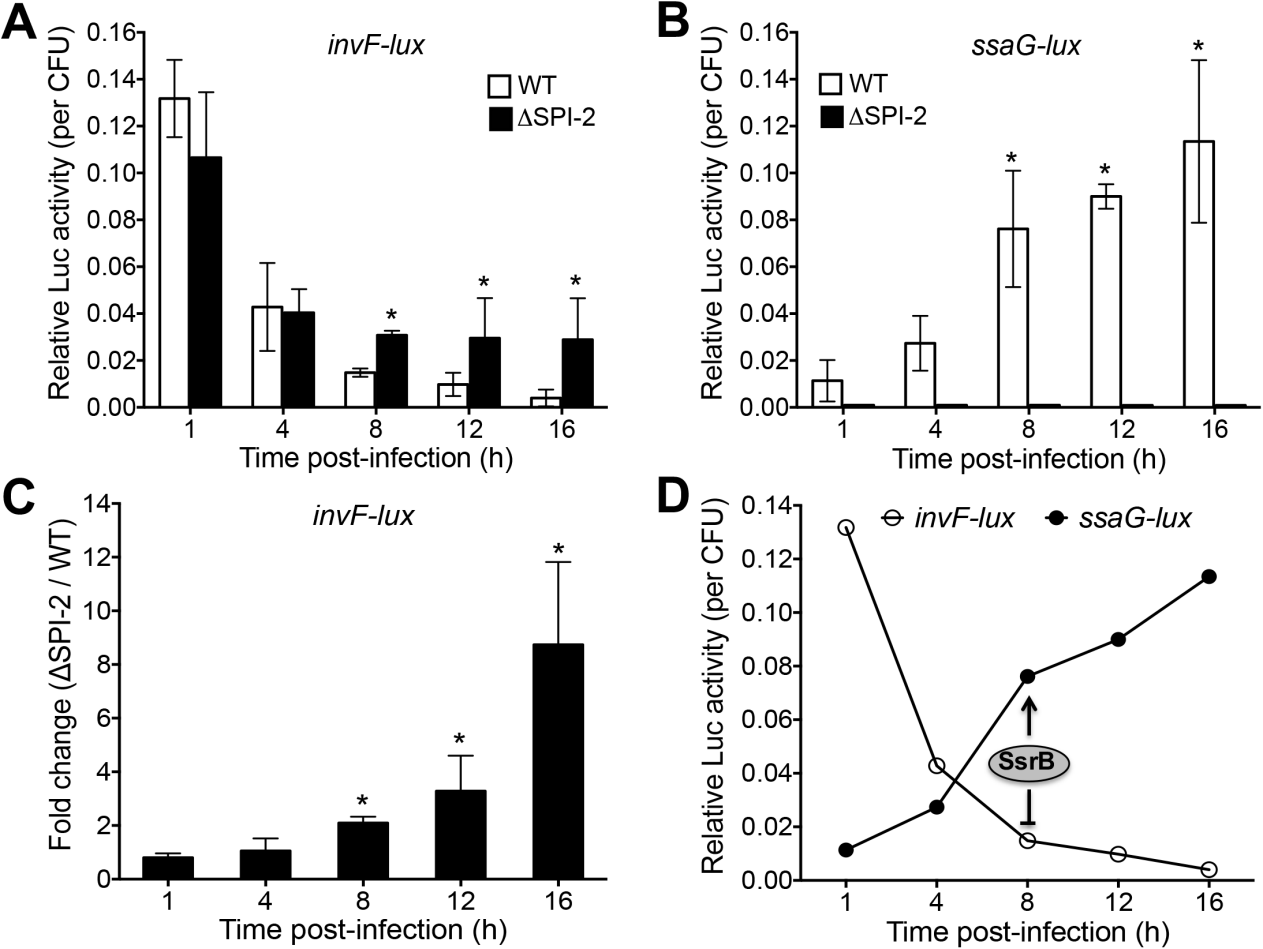
SsrB inversely regulates the expression of the *invF* (SPI-1) and *ssaG* (SPI-2) genes inside macrophages. Expression of the *invF-lux* (A) and *ssaG-lux* (B) transcriptional fusions was analyzed in the WT *S*. Typhimurium strain and its isogenic ΔSPI-2 mutant (lacking SsrB) inside RAW264.7 murine macrophage-like cells. Monolayers of macrophages were infected with an equal number of the respective *Salmonella* strain. At the indicated times post-infection the cells were lysed and luminescence and CFU counts were determined. Data represents the mean with standard deviation of three independent experiments. *Statistically different values with respect to those shown by the *invF-lux* fusion in the WT strain at the same post-infection times in panel *A* or with respect to that shown by the *ssaG-lux* fusion in the WT strain at 1 h post-infection in panel *B*, *P* < 0.05. (C) Data used in panel *A* were graphed to show the fold change in the expression of *invF-lux* in the ΔSPI-2 mutant with respect to the WT strain at the different post-infection times. *Statistically different values with respect to those obtained for 1 h post-infection, *P* < 0.05. (D) Data used in panels A and B were graphed to show the expression of the *invF-lux* and *ssaG-lux* fusions in the WT strain at the different post-infection times. Positive (indicated by an arrow) and negative (denoted by a blunt-end line) SsrB-mediated regulation of *ssaG* and *invF*, respectively, is depicted.

RAW264.7 macrophages were infected with WT *S*. Typhimurium or its isogenic ΔSPI-2 mutant carrying the *invF-lux*, *ssaG-lux* or *hns-lux* fusions. At specific time points after infection the macrophages were lysed and luminescence was measured and normalized to the number of viable intracellular bacteria. As expected, the intracellular replication of the WT strain increased over time whereas the ΔSPI-2 mutant decreased (S6 Fig). The intracellular expression of *invF-lux* and *ssaG-lux* also changed as expected in the WT strain, where *invF* expression decreased fifteen-fold by the last time point and *ssaG* expression increased the same magnitude over the course of the infection (Fig 8A and 8B). When comparing the expression levels of *invF-lux* between the WT strain and the ΔSPI-2 mutant, two distinct stages were identified. At 1 and 4 h post-infection, the *invF-lux* fusion showed similar expression levels in the WT strain and the ΔSPI-2 mutant, including a decrease in expression at 4 h (Fig 8A). However, at later time points in the infection, *invF-lux* expression levels continued to decrease in the WT strain, by two to nine-fold, but not in the ΔSPI-2 mutant (Fig 8A and 8C). This revealed SsrB-dependent repression of *invF* during intracellular infection. Furthermore, the *hns-lux* transcriptional fusion showed similar levels of intracellular expression in the WT and ΔSPI-2 strains at all time points of the infection (S7 Fig). Thus, the differences in the intracellular expression levels shown by the *invF-lux* fusion in the WT strain and its derivative ΔSPI-2 mutant were not due to the different levels of intracellular bacteria at these time points. On the other hand, only background activity was detected for the *ssaG-lux* fusion in the ΔSPI-2 mutant (Fig 8B), consistent with its expression being dependent on SsrB [3]. Interestingly, de-repression of the *invF-lux* intracellular expression in the ΔSPI-2 mutant coincided with the timing of induction of the *ssaG-lux* intracellular expression in the WT strain (Fig 8A and 8B). As expected, the de-repression of the *invF-lux* intracellular expression was also evident in a Δ*ssrA* and a Δ*ssrB* mutant, whereas the expression of the *hns-lux* control fusion was similar in the WT strain and these two mutants (S8 Fig), which indicates that both the SsrA sensor kinase and SsrB response regulator are required for intracellular repression of *invF* and that no other SPI-2-encoded factors are required. Together, these results show that SsrB simultaneously represses and induces the expression of *invF* and *ssaG*, respectively, inside macrophages (Fig 8D). Therefore, our data support that SsrB is involved in a regulatory switch that helps to coordinate the intracellular reprogramming of *Salmonella* genes, by activating the genetic program required for intracellular survival while de-activating the genes involved in the now-completed invasion step of infection.

### SsrB negatively regulates SPI-1 during mouse infections

**Fig 9:**
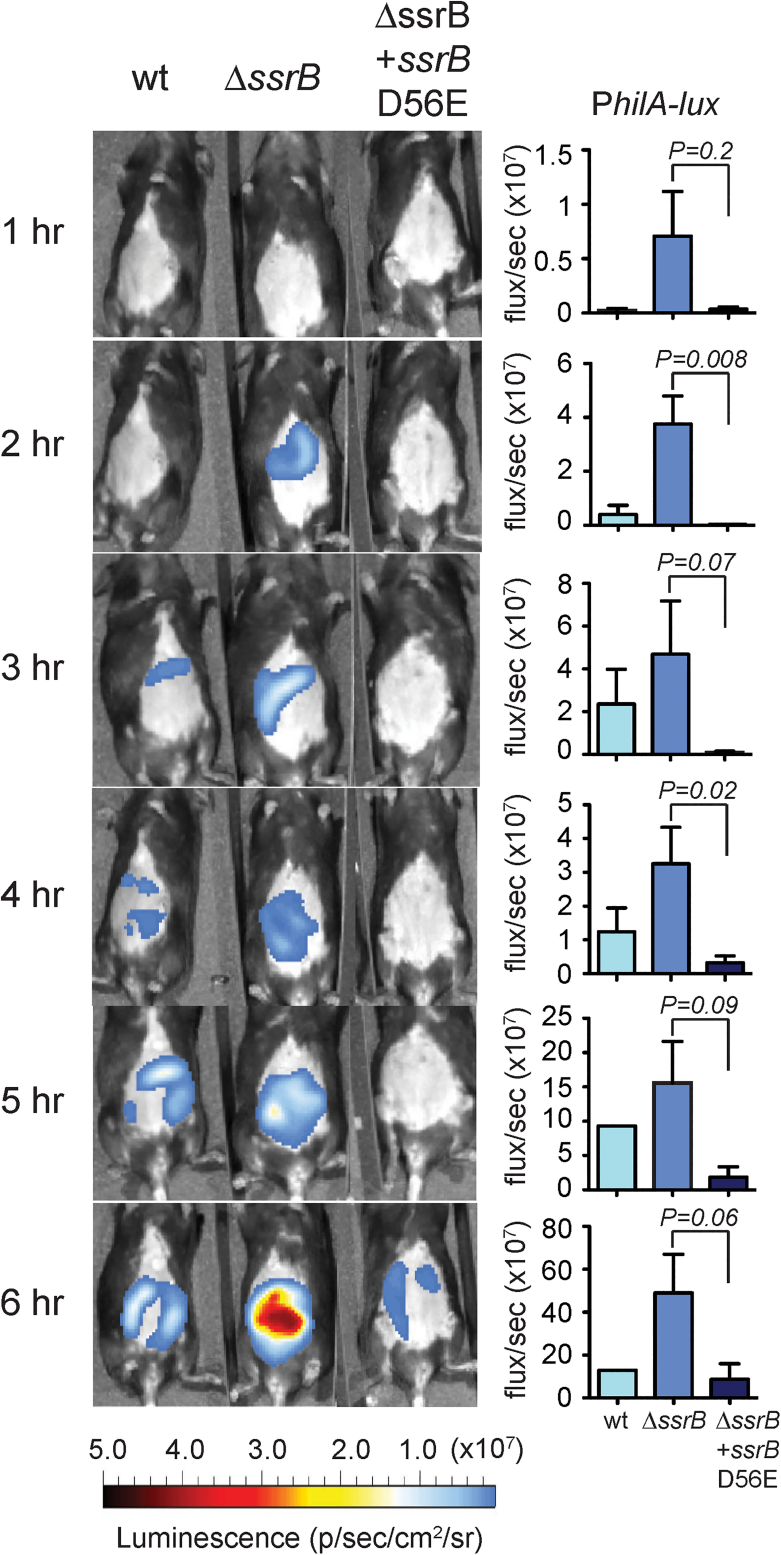
SsrB represses *hilA in vivo*. Mice were orally gavaged with the indicated strains and luciferase activity expressed from the *hilA-lux-740+350* fusion was measured by live animal imaging. Images are representative of three experiments and data is shown as the mean with standard error at each time point from three separate animals.

To determine whether SsrB represses expression of SPI-1 during mouse infections, we tested the *hilA-lux-740+350* transcriptional fusion in the WT *S*. Typhimurium strain, its isogenic Δ*ssrB* mutant, and in the Δ*ssrB* mutant complemented with a constitutive active SsrB variant in which aspartic acid 56 was replaced with glutamic acid. This SsrB D56E variant was expressed from the native *ssrA* promoter (P*ssrA-ssrB* D56E). C57BL/6 mice were orally gavaged with these strains and luminescence was quantified by *in vivo* imaging ever h for 6 h post-infection. Expression of the *hilA-lux* fusion was greater in the Δ*ssrB* mutant than in the WT strain at the different times tested, which was evident by quantification of total abdominal luminescence (Fig 9). The presence of SsrB D56E reduced the expression of the *hilA-lux* fusion in the Δ*ssrB* mutant (Fig 9). These results show that SsrB negatively regulates SPI-1 during the course of the intestinal infection of *S*. Typhimurium in a mouse model.

## Discussion

**Fig 10:**
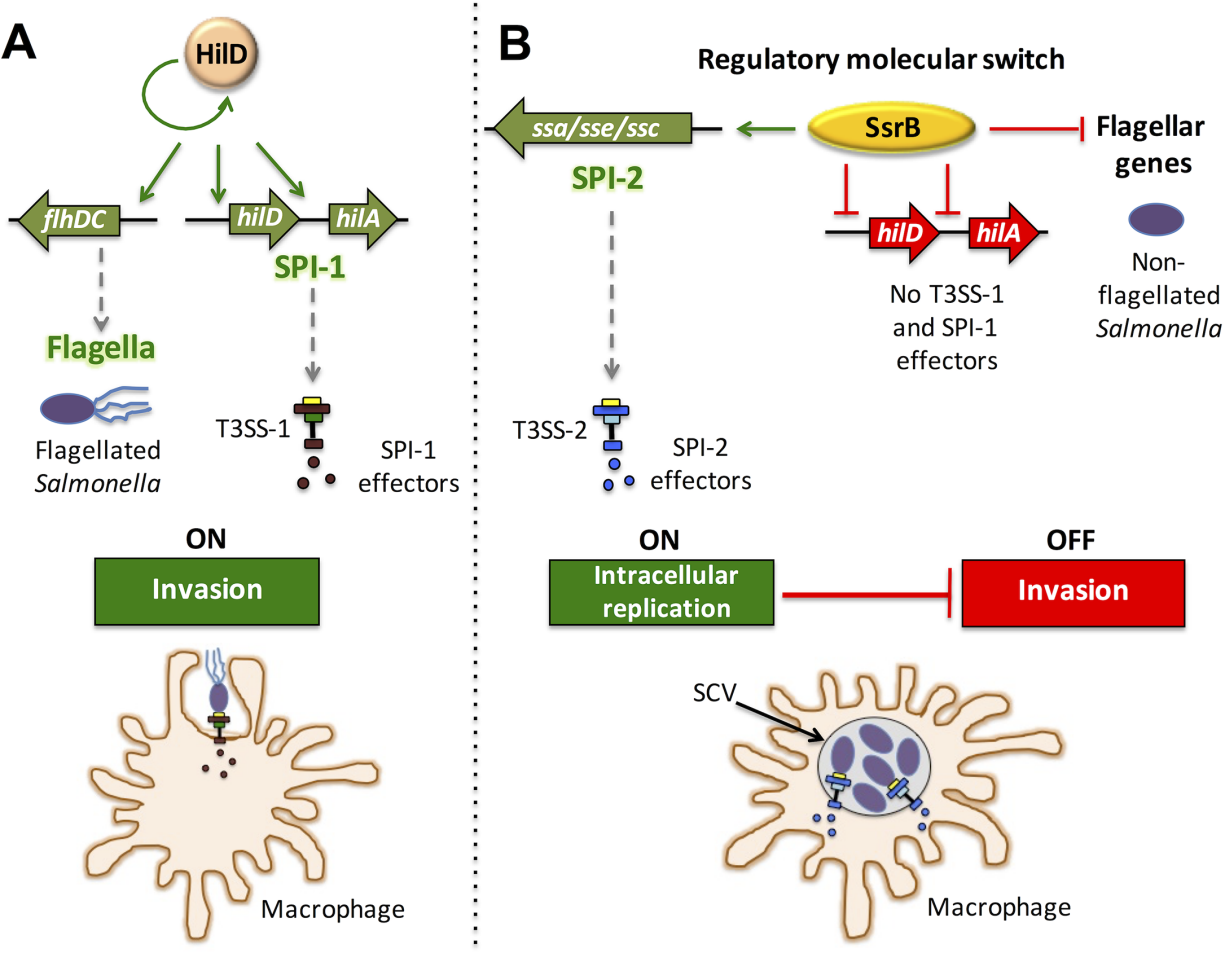
SsrB is involved in a molecular regulatory switch that aids in *Salmonella* transition to an intracellular lifestyle. (A) HilD directly or indirectly activates the expression of the SPI-1 genes and several other genes located outside SPI-1, including the flagellar regulatory operon *flhDC* required for the invasion of host cells. (B) Following its uptake into macrophages, *Salmonella* resides inside vacuoles, where SsrB induces the expression of the SPI-2 genes and other genes located outside SPI-2, which are required for survival and replication, while simultaneously repressing the expression of the *hilD* and *hilA* SPI-1 regulatory genes, and the flagellar-based motility genes. Green arrows and red blunt-end lines indicate positive and negative control, respectively, whereas gray dashed arrows denote expression of the respective genes.

*Salmonella* has developed a complex regulatory network to express virulence genes in a highly coordinated manner within particular host niches. For example, when *Salmonella* is inside macrophages, it down-regulates the SPI-1 invasion machinery and flagellar-based motility genes that are required for host-cell invasion, whereas the expression of the SPI-2 genes required for intracellular survival and replication is activated [9–11, 31]. Previously, the mechanism responsible for repressing the genes involved in invasion following successful invasion events was not known. Here, we show that this mechanism involves the SsrB response regulator, which had previously known roles in activating genes required for intracellular survival. Our data support a model in which SsrB acts as a key component of the molecular switch that helps *Salmonella* transition from an extracellular to an intracellular lifestyle (Fig 10). Interestingly, in a previous study it was demonstrated that SsrB, in its unphosphorylated form, drives a *Salmonella* lifestyle switch by relieving biofilm silencing [58].

Recent transcriptomics and proteomics data support that SsrB represses the expression of the SPI-1 and flagellar associated genes in *in vitro* SPI-2-inducing growth conditions [17, 32], and that it represses the flagellar genes when *S*. Typhimurium is inside macrophages [17]. In *S*. Typhi, a human-restricted serovar that causes systemic infections, the transcriptional regulator TviA represses the expression of the SPI-1 and flagellar genes and reduces macrophage pyroptosis [33–36]; pyroptosis and apoptosis are programmed cell death pathways stimulated by SPI-1 and flagellar gene products [37–39]. Interestingly, *S*. Typhimurium lacks the TviA regulator, which implied the existence of a different pathway in non-typhoidal serovars of *Salmonella*. The SsrB-mediated repression of the SPI-1 and flagellar genes in *S*. Typhimurium might be important in order to limit pyroptosis and apoptosis following infection by this serotype. Although we have not yet examined the impact of SsrB-mediated repression of invasion genes on these host cell pathways, the mechanism uncovered here may serve to limit damage to host cells as *Salmonella* establishes a stable intracellular niche.

Our data strongly support a mechanism whereby SsrB represses the SPI-1 genes by directly acting on the *hilD* and *hilA* regulatory genes. The direct binding of SsrB to the promoter of *hilD* may be preventing RNA polymerase from binding to this region. In addition to reducing the levels of HilD, SsrB-binding to the sequence centered at position -70 of *hilA*, overlapping a HilD-binding site, inhibits the HilD-mediated expression of *hilA*. These findings provide further insight on the SsrB regulon, and demonstrate how SsrB can act as a negative transcriptional regulator, in addition to its well-known role as a transcriptional activator. Moreover, previous studies indicate that the regulation of the SPI-1 genes mostly involves the control of *hilD* at the post-transcriptional and post-translational level [3, 24, 40]. Our results reveal another pathway for the regulation of SPI-1 that involves repression of *hilD* and *hilA* at the transcriptional level.

In *Escherichia coli*, the EnvZ-OmpR two-component system responds to osmotic stress signals [41]. The inverse regulation of the SPI-1 and SPI-2 genes by SsrB resembles the reciprocal control of *ompC* and *ompF* transcription by OmpR. OmpR is known to directly activate expression *of ssrA-ssrB*, and repress the expression of *hilD* [42, 43]. In addition to OmpR, other regulators, such as SlyA and PhoP, also positively and negatively control the expression of SPI-2 and SPI-1 genes, respectively [3, 11, 32, 43, 44]. Notably, OmpR, SlyA and PhoP positively control the expression of SsrB [3]. Therefore, these regulators may provide additional input into the SsrB-dependent or independent mechanisms that inversely regulates the expression of the SPI-1 and SPI-2 genes within macrophages.

In a previous study, we found that HilD mediates transcriptional crosstalk between SPI-1 and SPI-2 when *S*. Typhimurium is grown in LB, through growth-phase dependent activation of HilA and SsrB [14]. Here, we demonstrate that SsrB represses the expression of HilD and HilA, and thus the SPI-1 genes, revealing that the transcriptional communication between SPI-1 and SPI-2 is bi-directional. The degenerate palindromic sequence motif that SsrB recognizes on DNA [20] may make this response regulator particularly suited to dual-level control of gene expression. For example, the flexibility in the SsrB binding site may allow the bacterium to sample a wide array of new regulatory connections that can then be further optimized and selected by *cis*-regulatory evolution.

## Materials and methods

### Ethics statement

Animal experiments were conducted according to guidelines set by the Canadian Council on Animal Care, using protocols approved by the Animal Review Ethics Board at McMaster University under Animal Use Protocol #13-07-20.

### Media and culture conditions

Bacterial cultures were grown at 37ΔC in LB containing 1% tryptone, 0.5% yeast agar and 1% NaCl, pH 7.5; in N-minimal medium (N-MM) containing 5 mM KCl, 7.5 mM (NH_4_)_2_SO_4_, 0.5 mM K_2_SO_4_, 1mM KH_2_PO_4_, 100 mM Tris-HCl (pH 7.5), 10 μM MgCl_2_ and 0.1% casamino acids; or in phosphate-carbon-nitrogen (PCN) minimal medium containing 80 mM MES (pH 5.8), 4 mM Tricine, 100 μM FeCl_3_, 376 μM K_2_SO_4_, 50 mM NaCl, 0.4 mM K_2_HPO_4_/KH_2_PO_4_ (pH 5.8), 0.4% glucose, 15 mM NH_4_Cl, 1 mM MgSO_4_, 10 μM CaCl_2_ and micronutrients (10 nM Na_2_MoO_4_, 10 nM Na_2_SeO_3_, 4 nM H_3_BO_3_, 300 nM CoCl_2_, 100 nM CuSO_4_, 800 nM MnCl_2_, 1 nM ZnSO_4_). When necessary, media were supplemented with ampicillin (200 μg ml^-1^), kanamycin (30 μg ml^-1^) or streptomycin (100 μg ml^-1^). Cultures in LB, N-MM or PCN media for chloramphenicol acetyltransferase (CAT) or Western blot assays were performed as described previously [12, 14, 45]. Briefly, overnight cultures of the *Salmonella* strains were sub-cultured (1:50) into 50 ml of fresh medium contained in 250 ml flaks, which were incubated at 37°C with shaking (200 r.p.m.) in an Orbital shaker bath (GYROMAX 902, Amerex Instruments), during the indicated times.

### Construction of mutant strains and strains expressing FLAG-tagged proteins

Bacterial strains used in this work are listed in Table 1. Deletion of *rtsA* in *S*. Typhimurium SL1344 was performed by the λ Red recombinase system, as described previously [46], using the primers shown in Table 2, generating the strain DTM91. P22 transduction was used to transfer the *invF*::*3XFLAG-kan* allele from strain DTM76 into *S*. Typhimurium SL1344, generating the strain DTM85, to transfer the Δ*ssrB*::*kan* allele from the strain MJW112 into the strain DTM86, generating the strain DTM87, to transfer the ΔSPI-1::*kan* allele from the strain ΔSPI-1 into DTM92, generating the strain DTM93, to transfer the Δ*Cthns*::*kan* allele from the strain DTM84 into the strain DTM94, generating the strain DTM95, to transfer the Δ*ssrB*::*kan* allele from the strain 4/74 Δ*ssrB* into *S*. Typhimurium SL1344, generating the strain DTM97, and to transfer the Δ*ssrA*::*kan* allele from the strain 4/74 Δ*ssrA* into *S*. Typhimurium SL1344, generating the strain DTM98. The kanamycin resistance cassette was excised from the strains DTM85, ΔSPI-2::*kan*, JPTM30, DTM91, DTM93, DTM95, DTM97 and DTM98, by using helper plasmid pCP20 expressing the FLP recombinase, as described previously [46], generating the strains DTM86, DTM89, DTM90, DTM92, DTM94, DTM96, DTM99 and DTM100, respectively. All mutant strains were verified by PCR amplification and sequencing.

**Table 1:** Bacterial strains and plasmids.

Name	Genotype	Reference
**Bacterial strains**	** **	** **
*S*. Typhimurium		
SL1344	Wild type; *xyl*, *hisG*, *rpsL*; Sm^R^	[53]
MJW112	Δ*ssrB*::*kan*	M. Worley and F. Heffron
ΔSPI-2	ΔSPI-2::*kan*	[54]
JPTM25	Δ*hilD*	[45]
JPTM30	*ssrB*::*3XFLAG-kan*	[45]
DTM76	14028s *invF*::*3XFLAG-kan*	[26]
DTM84	14028s Δ*hilD* Δ*Cthns*::*kan*	[26]
DTM85	*invF*::*3XFLAG-kan*	This study
DTM86	*invF*::*3XFLAG*	This study
DTM87	Δ*ssrB*::*kan invF*::*3XFLAG*	This study
DTM88	Δ*flhDC*::*kan*	[55]
DTM89	ΔSPI-2	This study
DTM90	*ssrB*::*3XFLAG*	This study
ΔSPI-1	ΔSPI-1::*kan*	[31]
DTM91	Δ*rtsA*::*kan*	This study
DTM92	Δ*rtsA*	This study
DTM93	Δ*rtsA* ΔSPI-1::*kan*	This study
DTM94	Δ*rtsA* ΔSPI-1	This study
DTM95	Δ*rtsA* ΔSPI-1 Δ*Cthns*::*kan*	This study
DTM96	Δ*rtsA* ΔSPI-1 Δ*Cthns*	This study
4/74 Δ*ssrB*	Δ*ssrB*::*kan*	[32]
4/74 Δ*ssrA*	Δ*ssrA*::*kan*	[32]
DTM97	Δ*ssrB*::*kan*	This study
DTM98	Δ*ssrA*::*kan*	This study
DTM99	Δ*ssrB*	This study
DTM100	Δ*ssrA*	This study
*E*. *coli*		
BL21/DE3	Strain for expression of recombinant proteins	Invitrogen
DH10β	Laboratory strain	Invitrogen
**Plasmids**	** **	** **
pKK232-8	pBR322 derivative containing a promotorless chloramphenicol acetyltransferase (*cat*) gene, Ap^R^	[56]
philD-cat-364+88	pKK232-8 derivative containing a *hilD-cat* transcriptional fusion from nucleotides -364 to +88	[14]
philD-cat-108+88	pKK232-8 derivative containing a *hilD-cat* transcriptional fusion from nucleotides -108 to +88	This study
philD-cat-48+88	pKK232-8 derivative containing a *hilD-cat* transcriptional fusionfrom nucleotides -48 to +88	This study
philD-cat-37+6	pKK232-8 derivative containing a *hilD-cat* transcriptional fusion from nucleotides -37 to +6	This study
philA-cat-410+446	pKK232-8 derivative containing a *hilA-cat* transcriptional fusion from nucleotides -410 to +446	[14]
philA-cat-410+66	pKK232-8 derivative containing a *hilA-cat* transcriptional fusion from nucleotides -410 to +66	This study
philA-cat-100+6	pKK232-8 derivative containing a *hilA-cat* transcriptional fusion from nucleotides -100 to +6	This study
philA-cat-100+6 Mut	*hilA-cat-*100+6 transcriptional fusion carrying mutations in the SsrB binding site	This study
philA-cat-35+6	pKK232-8 derivative containing a *hilA-cat* transcriptional fusion from nucleotides -35 to +6	This study
philA-cat-35+446	pKK232-8 derivative containing a *hilA-cat* transcriptional fusion from nucleotides -35 to +446	This study
pinvF-cat	pKK232-8 derivative containing a *invF-cat* transcriptional fusion from nucleotides -306 to +231	[14]
pssaG-cat	pKK232-8 derivative containing a *ssaG-cat* transcriptional fusion from nucleotides -303 to +361	[14]
psirA-cat	pKK232-8 derivative containing a *sirA-cat* transcriptional fusion from nucleotides -563 to +98	[45]
pcsrA-cat	pKK232-8 derivative containing a *csrA-cat* transcriptional fusion from nucleotides -327 to +61	[45]
pfliC-cat	pKK232-8 derivative containing a *fliC-cat* transcriptional fusion from nucleotides -220 to +160	This study
pCS26-*Pac*	pZS derivative containing a promoterless *luxCDABE* operon, Kan^R^	[47]
pinvF-lux	pCS26-*Pac* derivative containing a *invF-lux* transcriptional fusion from nucleotides -306 to +231	This study
pssaG-lux	pCS26-*Pac* derivative containing a *ssaG-lux* transcriptional fusion from nucleotides -303 to +361	This study
phns-lux	pCS26-*Pac* derivative containing a *hns-lux* transcriptional fusion from nucleotides -967 to +73	This study
pGEN-l*uxCDABE*	p15A derivative low-copy-number plasmid carrying the *luxCDABE* operon downstream the	[48]
	constitutive *em7* synthetic promoter, Ap^R^	
philA-lux-740+350	pGEN-*luxCDABE* derivative containing a *hilA-lux* transcriptional fusion from nucleotides -740 to +350	This study
philA-lux-36+446	pGEN-*luxCDABE* derivative containing a *hilA-lux* transcriptional fusion from nucleotides -36 to +446	This study
pCP20	Plasmid expressing FLP recombinase from a temperature-inducible promoter, Ap^R^	[46]
pMPM-K3	Low-copy-number cloning vector, p15A *ori*, *lac* promoter, Kan^R^	[49]
pK3-SsrB	pMPM-K3 derivative expressing SsrB from the *lac* promoter	This study
pWSK129	Low-copy-number cloning vector, pSC101 *ori*, Kan^R^	[57]
pP*ssrA-ssrB* (D56E)	pWSK129 derivative expressing SsrB with the D56E mutation from the native *ssrA* promoter	This study
pK6-HSsrBc	pMPM-K6Ω derivative expressing 6H-SsrBc from an arabinose-inducible promoter, Kan^R^	M.A. De la Cruz
pMAL-HilD1	pMAL-c2X derivative expressing MBP-HilD from a *lac* promoter, Ap^R^	[14]

The coordinates for the *cat* and *lux* fusions are indicated with respect to the transcriptional start site for each gene. Ap^R^, ampicillin resistance; Kan^R^, kanamycin resistance; Sm^R^, streptomycin resistance.

**Table 2:** Oligonucleotides.

Primer	Sequence (5`-3`)	Target gene	*RE
**For *cat* transcriptional fusions and EMSAs**			
hilD-108FW	AACGGATCCAGATAAATTACCCAAATTTGGGTTC	*hilD*	BamHI
hilD-48FW	CAAGGATCCCTAATAAAGAGCATTTACAACTCAG	*hilD*	BamHI
hilDRHindIII(rv)	CTGAAGCTTATCTGCGGCAGGACGC	*hilD*	HindIII
hilDPFBam(fw)	GATCCGCATTTACAACTCAGATTTTTTCAGTAGGATACCAGTAAGGA	*hilD*	BamHI
hilDPRHind(rv)	AGCTTCCTTACTGGTATCCTACTGAAAAAATCTGAGTTGTAAATGCG	*hilD*	HindIII
hilA1FBam(fw)	ATCGGATCCCTCTGAGAACTATTTGC	*hilA*	BamHI
hilAp+66H(rv)	CAGAAGCTTTCAGCGCCGGGCATC	*hilA*	HindIII
hilA-100Ba(fw)	TAGGGATCCTCTTCGAGAAAAATGGTTCTG	*hilA*	BamHI
hilA+6Hind(rv)	AGAAAGCTTTTTTGTAGCTATCTTACTGC	*hilA*	HindIII
hilAPFBam(fw)	GATCCGCATTTACACCCCAAAAAAATGCAGTAAGATAGCTACAAA	*hilA*	BamHI
hilAPRHind(rv)	AGCTTTTGTAGCTATCTTACTGCATTTTTTTGGGGTGTAAATGCG	*hilA*	HindIII
hilAp+66FB(fw)	TAAGGATCCGCATTTACACCCCAAAAAAATG	*hilA*	BamHI
hilA2RHind(rv)	GACAAGCTTTTCTGAGCGTAGCAGGG	*hilA*	HindIII
hilA-100MutFw	TAGGGATCCTCTTCGAGAAAAATGGTTCTGGGGGTGTAATTTTGAGGCCATTAACCATGA		
fliC-RVI-BH	GTTGGATCCCACACCTAATGATG	*fliC*	BamHI
fliC-FWI-Hd	GACAAGCTTACAGACGCTCGATAGCGGTG	*fliC*	HindIII
**For *lux* transcriptional fusions**			
invF-luxR1	GATGGATCCGCGACAACGGCCTGCTCGC	*invF*	BamHI
invF-luxF2	ATCCTCGAGCAGAAGAATGAGGCGCCATG	*invF*	XhoI
ssaG-luxR1	ATCGGATCCAACAATAACCGTTAGCGCTGG	*ssaG*	BamHI
ssaG-luxF2	ATTCTCGAGGAGTGGTAGTTTGGGACTAC	*ssaG*	XhoI
hns-luxR1	CCTGGATCCGAAGAGTACGGATGTTGTTC	*hns*	BamHI
hns-luxF2	GCTCTCGAGACCATGCCAGCAAGTATTGG	*hns*	XhoI
EC30F	CGGCGGATCCCATTTTTTGTATCTGTCACTTAAGT	*hilA*	BamHI
EC30R	CGCCTACGTAGATAATAGTGTATTCTCTTACAGGG	*hilA*	SnaBI
EC76F	CGGCGGATCCGCATTTACACCCCAAAAAAATGCAG	*hilA*	BamHI
EC77R	CGGCTACGTACTTTTCTGAGCGTAGCAGGG	*hilA*	SnaBI
**For gene cloning**			
SRBF19-KpnI	GCGGGTACCGAACTAACCGACTTACG	*ssrB*	KpnI
ABR15-SacI	TGGGAGCTCATACCAGGGCATCCGTATGG	*ssrB*	SacI
DTM17F-SalI	ACGCGTCGACAAATGGAGTTTCTATCAAA	P*ssrA*	SalI
DTM17.2R	AATGCTTCCCTCCAGTTGCC	P*ssrA*	
DTM17.1F	GGCAACTGGAGGGAAGCATTATGAAAGAATATAAGATCTT	*ssrB*	
DTM17R-XbaI	GCTCTAGATTAATACTCTATTAACCTCA	*ssrB*	XbaI
DTM299F	CATACGAGCCTGACATACTTATCCTTGAACTTAGTCTACCTGGCATCAATGGCC	*ssrB* D56E	
DTM299R	GGCCATTGATGCCAGGTAGACTAAGTTCAAGGATAAGTATGTCAGGCTCGTATG	*ssrB* D56E	
**For gene deletions**			
rtsA-H1P1	TAATAAAAGGAAATTATCATGCTAAAAGTATTTAATCCCTCA*TGTAGGCTGGAGCTGCTTCG*	*rtsA*	
rtsA-H2P2	TTGATGACGAGAGGAAGATAAAAACGCTAAAAATTCCGATGG*CATATGAATATCCTCCTTAG*	*rtsA*	

*RE, restriction enzyme for which a site was generated in the primer. Underlined letters indicate the respective restriction-enzyme site in the primer. Italic letters show the sequences corresponding to the template plasmid pKD4.

### Construction of plasmids

Plasmids and primers used in this work are listed in Tables 1 and 2, respectively. To construct the plasmids containing the transcriptional fusions *hilD-cat-108+88*, *hilD-cat-48+88*, *hilA-cat-410+66*, *hilA-cat-100+6*, *hilA-cat-100+6* Mut, *hilA-cat-35+446* and *fliC-cat*, the respective segment of the regulatory region of *hilD*, *hilA* or *fliC* were amplified by PCR with the primer pairs hilD-108FW/hilDRHindIII(rv), hilD-48FW/hilDRHindIII(rv), hilA1FBam(fw)/hilAp+66H(rv), hilA-100Ba(fw)/hilA+6Hind(rv), hilA-100MutBamH(fw)/hilA+6Hind(rv), hilAp+66FB(fw)/hilA2RHind(rv) or fliC-RVI-BH/fliC-FWI-Hd. The PCR products were digested with BamHI and HindIII restriction enzymes and then cloned into the BamHI and HindIII sites of the vector pKK232-8, which carries a promotorless *cat* gene (Amersham Pharmacia LKB Biotechnology), generating plasmids philD-cat-108+88, philD-cat-48+88, philA-cat-410+66, philA-cat-100+6, philA-cat-35+446 and pfliC-cat. To construct the plasmids containing the transcriptional fusions *hilD-cat-37+6* and *hilA-cat-35+6*, the complementary primers hilDPFBam(fw) and hilDPRHind(rv) or hilAPFBam(fw) and hilAPRHind(rv), each at a final concentration of 50 μM, were annealed by heating them together at 94°C for 10 min and then slowly cooling to room temperature. The obtained double-strand products carried cohesive ends for their cloning into the BamHI and HindIII sites of the vector pKK232-8, generating plasmids philD-cat-37+6 and philA-cat-35+6. To construct the plasmids containing the transcriptional fusions *invF*-*lux*-306+231, *ssaG*-*lux*-303+361 and *hns*-*lux*-967+73, the respective segment of the regulatory region of *invF*, *ssaG* or *hns* were amplified by PCR with the primer pairs invF-luxR1/invF-luxF2, ssaG-luxR1/ssaG-luxF2 or hns-luxR1/hns-luxF2, respectively. The PCR products were digested with BamHI and XhoI enzymes and then cloned into the same restriction sites of the pCS26-*Pac* vector, which carries a promotorless *lux* operon [47], generating the p*invF*-*lux*, p*ssaG*-*lux* and p*hns*-*lux* plasmids. The *hilA*-*lux-740+350* and *hilA*-*lux-36+446* transcriptional fusions were constructed by replacing the *em7* promoter in the pGEN-*luxCDABE* plasmid [48] (Addgene plasmid # 44918) with the respective regulatory region of *hilA*. The regulatory region of *hilA* was amplified by PCR with the primer pairs EC30F/EC30R or EC76F/EC77R. The PCR products were digested with BamHI and SnaBI enzymes and then cloned into the same restriction sites of the pGEN-*luxCDABE*, generating the p*hilA*-*lux-740+350* and p*hilA*-*lux-36+446* plasmids. To construct the pK3-SsrB plasmid, the *ssrB* gene was amplified by PCR using the primer pair SRBF19-KpnI/ABR15-SacI and chromosomal DNA from the WT *S*. Typhimurium SL1344 as template. The PCR products were digested with KpnI and SacI restriction enzymes and then cloned into the vector pMPM-K3 [49] digested with the same restriction enzymes. The pK3-SsrB plasmid constitutively expresses SsrB from a *lac* promoter, since *Salmonella* and the vector pMPM-K3 lack the gene encoding LacI, the repressor of *lac*. The constitutively active SsrB variant (P*ssrA-ssrB* D56E) was generated by cloning the *ssrA* promoter (amplified with primers DTM17F/17.2R) and *ssrB* coding sequence (primers DTM17.1F/17R) from *S*. Typhimurium SL1344 into pBluescript using SOE PCR. SDM was performed on this plasmid in pBluescript with primers DTM299F/299R to generate P*ssrA-ssrB* (D56E). This was subsequently subcloned into the low copy vector pWSK129 using the SalI and XbaI restriction sites.

### Protein secretion analysis and Western blotting

Protein secretion and Western blot assays were performed as we described previously [45]. Immunoblots were performed with anti-FLAG M2 (Sigma) or anti-DnaK (StressGen) monoclonal antibodies at 1:4,000 and 1:20,000 dilutions, respectively. Horseradish peroxidase-conjugated anti-mouse (Pierce) at a dilution of 1:10,000 was used as the secondary antibody.

### CAT assays

The CAT assays and protein quantification to calculate CAT specific activities were performed as previously described [50].

### Expression and purification of 6H-SsrBc

*E*. *coli* BL21/DE3 containing pK6-HSsrBc was grown in 200 ml of LB at 37°C with shaking. At an optical density (OD_600_) of 0.6, expression of 6H-SsrBc was induced by adding 0.1% L-arabinose and cultures were incubated for an additional 4 h. Bacterial cells were harvested by centrifugation at 4°C and the 6H-SsrBc protein was purified from pellet as previously described [25].

### Expression and purification of MBP-HilD

Maltose binding protein (MBP)-HilD was expressed in *E*. *coli* BL21/DE3 containing pMAL-HilD1 and purified by using an amylose column, as described previously [14].

### Electrophoretic mobility shift assays (EMSAs)

Fragments of the regulatory regions of *hilD*, *hilA*, *invF*, *ssaG*, *sirA* and *csrA* were obtained by PCR amplification with the same primer pairs used to construct the respective transcriptional fusion to the *cat* reporter gene. PCR products were purified using the QIAquick PCR purification kit (Qiagen). Each PCR product (≈100 ng) was mixed with increasing concentrations of purified 6H-SsrBc in a binding buffer containing 10 mM Tris (pH 7.5), 50 mM KCl, 2.5% glycerol, 5 mM MgCl_2_ and 0.05% Nonidet P-40, in a final volume of 20 μl. Protein-DNA binding reactions were incubated at room temperature for 20 min; then separated by electrophoresis in 6% non-denaturing acrylamide gels in 0.5 X Tris-borate-EDTA buffer, at room temperature. The DNA fragments were stained with ethidium bromide and visualized with an Alpha-Imager UV transilluminator (Alpha Innotech Corp.).

### Invasion assays

Gentamicin protection assays were performed as previously described [22]. HeLa (human cervical adenocarcinoma epithelial) cells (ATCC) were grown in high-glucose Dulbecco´s Modified Eagle Medium (DMEM) (GIBCO 12100–046) supplemented with 10 mM sodium pyruvate solution (SIGMA S8636), 20 mM L-glutamine (GIBCO 25030–081) and 10% (v/v) heat-inactivated fetal bovine serum (ByProductos 13001), at 37°C in a humidified atmosphere with 5% CO_2_. HeLa cells were seeded 20 h prior to infection in 24-well tissue culture plates at 1 x 10^5^ cells per well. Overnight *Salmonella* cultures were sub-cultured 1:33 in 20 ml of fresh LB and incubated at 37°C with shaking for 4 h. The sub-cultures were diluted (1:5) in LB to OD_600_ of 0.6. At this point, 1 ml of each sub-culture was spun and resuspended in 1 ml of 1X PBS. Then, 10 μl of these bacterial suspensions were used to infect the HeLa cells at a multiplicity of infection (MOI) of 30:1 (bacteria to eukaryotic cell) for 10 min. Cells were then washed twice with pre-warmed 1X PBS and incubated for an additional 20 min with DMEM at 37°C. Following this incubation time, monolayers were incubated with DMEM containing 50 μg/ml gentamicin for 1 h to eliminate any extracellular bacteria. The media was then removed and the cells were lysed in 1 ml of 0.2% (w/v) sodium deoxycholate in 1 X PBS. The cell lysates and the initial starting inoculums were serial diluted and plated onto LB agar supplemented with streptomycin at 100 μg ml^-1^.

### Bioluminescent reporter assays

Overnight cultures of the *Salmonella* strains containing the *hilA*-*lux* transcriptional fusions were sub-cultured (1:50) into LB broth at 37°C until the cultures reached mid-exponential phase (OD_600_ = 0.5). The cultures were sub-cultured (1:50) again into LB in black 96-well polystyrene plates. Plates were incubated at 37°C with shaking, and luminescence and OD_600_ were measured every 30 min using the PerkinElmer Plate Reader. Luminescence was normalized to OD_600_.

To determine intracellular gene expression using the *lux* bioluminescent reporter, we performed infection assays using RAW264.7 murine macrophage-like cells (ATCC), as described for the invasion assays with HeLa cells. The RAW264.7 cells were seeded at a density of 1.5 x 10^6^ cells/plate in 100 mm x 20 mm culture dishes (Corning 430167) and infected with the *Salmonella* strains carrying the *lux-*transcriptional fusions at an MOI of 10:1 (bacteria to eukaryotic cell). Following gentamicin treatment, the cells were lysed at 1, 4, 8, 12, and 16 h post-infection in 600 μl of 0.2% (w/v) sodium deoxycholate in 1 X PBS. A 200 μl sample of the cell lysates was loaded in duplicate into a white 96 well assay plate with a clear flat bottom (Corning 3610) and luminescence was measured using the GloMax-Multi Detection System (Promega). Cell lysates were also plated and luminescence was normalized to bacterial CFUs. Replication was determined by enumerating the recovered CFUs at 4, 8, 12, and 16 h post-infection. Fold-replication represents the CFUs recovered at 4, 8, 12 or 16 h relative to the CFUs at 1 h post-infection.

### *In vivo* bioluminescent imaging

One day prior to infection, C57BL/6 mice were orally gavaged with 20 mg of streptomycin and abdominal fur was removed using clippers and depilatory cream. The WT *S*. Typhimurium SL1344 strain and its isogenic Δ*ssrB* and Δ*ssrB* complemented with the pP*ssrA-ssrB* (D56E) plasmid, each containing the *hilA-lux-740+350* fusion, were grown overnight with shaking at 37°C in LB supplemented with 100 mg ml^-1^ ampicillin and 50 mg ml^-1^ kanamycin. Bacteria were washed twice in 0.1 M HEPES (pH 8) + 0.9% NaCl and mice were orally gavaged with 1x10^8^ CFUs. Following infection, mice were anaesthetized with 2% isoflourane carried in 2% oxygen and imaged dorsally in an IVIS Spectrum (PerkinElmer). Grey scale and luminescent images were captured every hour for six hours. Total abdominal luminescence was quantified at each time point.

### Bioinformatics analysis

Computational analyses were performed with the regulatory sequence analysis tools (RSAT) [51, 52]. The position-specific scoring matrix (PSSM) for the DNA-binding consensus sequence of SsrB was generated using the conserved 18 bp palindrome sequence that SsrB is known to recognize [20]. The prediction of SsrB-binding sites in the regulatory regions of *hilD* and *hilA* was performed with the matrix-scan program and the PSSM we created, using a *P*-value of 1e-3. Default parameters were used in these computational programs unless otherwise indicated.

### Statistical analysis

Data were analyzed with GraphPad Prism 5.0 software (GraphPad Inc., San Diego, CA) using unpaired Student`s *t*-test. For *in vivo* bioluminescence analyses, data outliers were identified using the Grubbs test. One data point was identified as an outlier and was omitted from the analysis in the WT (1 h) group.

## Supporting information

S1 Fig Negative controls for *cat* reporter assays and EMSAs. Expression of the transcriptional fusions *sirA-cat* (A) and *csrA-cat* (B) was determined in the WT *S*. Typhimurium strain containing the vector pMPM-K3, or the plasmid pK3-SsrB, which expresses SsrB from a constitutive promoter. The CAT-specific activity was determined from bacterial cultures grown for 4 and 9 h in LB at 37°C. Data represents the mean with standard deviation of three independent experiments. EMSAs were performed to examine whether SsrB binds to the DNA fragments in the *sirA-cat* (C) and *csrA-cat* (D) fusions. The DNA fragments were incubated with increasing concentrations of purified 6H-SsrBc (0, 0.5, 1, 1.5 and 2 σM). DNA-protein complexes are indicated by an asterisk.
(TIFF)

S2 Fig Repression of *hilA* by SsrB requires the DNA sequence located upstream of the *hilA* promoter. Expression of the *hilA-lux-740+350* (full length) (A) and *hilA-lux-36+446* (truncated) (B) transcriptional fusions was determined in the WT *S*. Typhimurium strain and its isogenic Δ*ssrB* mutant containing or not the pWSK129 vector, or the pP*ssrA-ssrB* (D56E) plasmid expressing SsrB with the D56E mutation, from its native promoter that is located upstream of *ssrA*. Luminescence (RLU) was quantified from bacterial cultures grown in LB at 37°C. RLUs were normalized to OD_600_ at each time point. Data represents the mean with standard deviation of three and two independent experiments for (A) and (B), respectively.
(TIFF)

S3 Fig Mutations in *hilA* affecting repression by SsrB also affect activation by HilD. Expression of the *hilA-cat-100+6* WT (WT SsrB binding site) (A) and *hilA-cat-100+6* Mut (mutated SsrB binding site) (B) fusions was determined in the WT *S*. Typhimurium strain and its isogenic Δ*hilD* mutant. The CAT-specific activity was determined from bacterial cultures grown for 9 h in LB at 37°C. Data represents the mean with standard deviation of three independent experiments. *Statistically different values relative to the WT strain, *P* < 0.0005. EMSAs were performed to analyze the interaction of HilD with the *hilA* DNA fragments carried by the *hilA-cat-100+6* WT (C) and *hilA-cat-100+6* Mut (D) fusions. The DNA fragments were incubated with increasing concentrations of purified MBP-HilD (0, 0.1, 0.5 and 1 μM). DNA-protein complexes are indicated by an asterisk.
(TIFF)

S4 Fig Comparison of the promoter sequences contained in the *hilD-cat-37+6* and *hilA-cat-35+6* transcriptional fusions. Common nucleotides are indicated by shading. The two predicted SsrB binding sites in *hilD* are shown by red letters. The transcriptional start site (+1) and the -35 and -10 promoter sequences are underlined.
(TIFF)

S5 Fig *invF* is not de-repressed in the absence of SsrB during *in vitro* growth conditions. (A) The expression of InvF-FLAG was analyzed by Western blot in the WT *S*. Typhimurium strain and in a Δ*ssrB* mutant, using monoclonal anti-FLAG antibodies. As a loading control, the expression of DnaK was also determined using monoclonal anti-DnaK antibodies. Expression of the *invF-cat* (B) and *ssaG-cat* (C) transcriptional fusions was measured in the WT and Δ*ssrB* strains with chromosomally FLAG-tagged *invF*. Data represents the mean with standard deviation of three independent experiments. *Statistically different values with respect to the WT strain are indicated, *P* < 0.0005. Expression of InvF-FLAG, and the *invF-cat* and *ssaG-cat* fusions was determined from bacterial cultures grown for 4 and 9 h in LB or at OD_600_ of 0.3 in PCN, at 37°C.
(TIFF)

S6 Fig Growth rates of the WT *S.* Typhimurium strain and its isogenic ΔSPI-2 mutant inside macrophages. Fold-replication represents the CFUs recovered at the different post-infection times relative to the CFUs at 1 h post-infection for each strain. The dashed line is used to distinguish between increased and decreased replication levels. Data represents the mean with standard deviation of three independent experiments.
(TIFF)

S7 Fig Expression of *hns* in macrophages is not affected by the absence of SsrB. The intracellular expression of the *hns-lux* transcriptional fusion was examined in the WT *S*. Typhimurium strain and its derivative ΔSPI-2 mutant (lacking SsrB) in RAW264.7 murine macrophage-like cells. Luminescence was quantified and normalized to CFU counts at 1, 4, 8, 12, and 16 h post-infection. The dashed line represents the relative luminescence per CFU of the WT *S*. Typhimurium strain with the promoterless pCS26-*Pac* vector in RAW264.7 cells. Data represents the mean with standard deviation of three independent experiments.
(TIFF)

S8 Fig SsrA and SsrB repress the expression of *invF* inside macrophages. Expression of the *invF-lux* (A) and *hns-lux* (B) transcriptional fusion was analyzed in the WT *S*. Typhimurium strain and its isogenic Δ*ssrA* and Δ*ssrB* mutants inside RAW264.7 murine macrophage-like cells. Monolayers of macrophages were infected with an equal number of bacteria of the respective *Salmonella* strain. At 16 h post-infection the cells were lysed and luminescence and CFU counts were determined as described in Materials and Methods. Data represents the mean with standard deviation of three independent experiments. *Statistically different values with respect to the WT strain, *P* < 0.005.
(TIFF)
