# Supplementary figures and images for: The transcriptional regulator SsrB is involved in a molecular switch controlling virulence lifestyles of Salmonella
Source: PLoS Pathog. 2017 Jul 13;13(7):e1006497. doi: 10.1371/journal.ppat.1006497 (PMC5562331; doi:10.1371/journal.ppat.1006497)

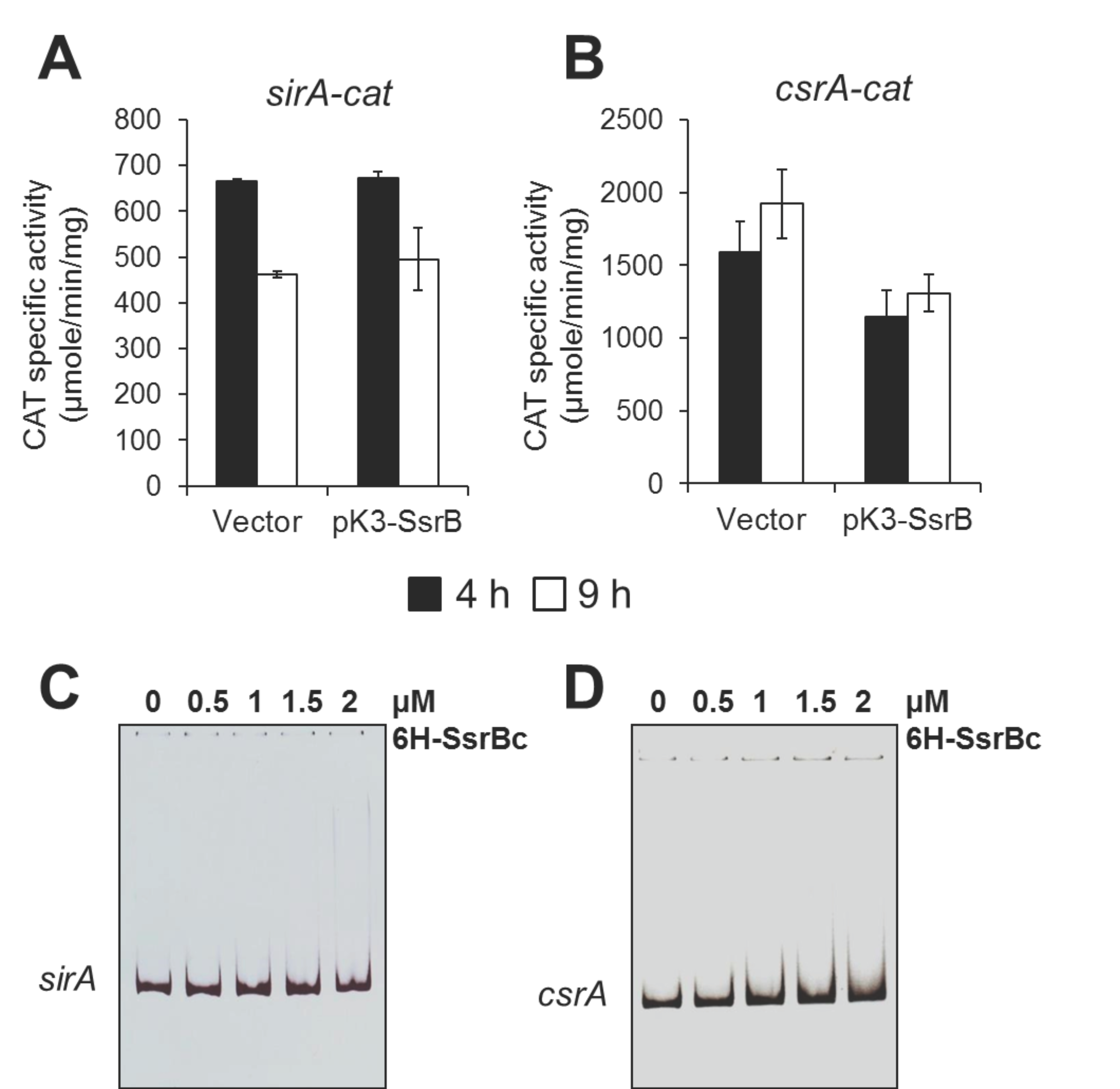

Supplement: S1 Fig — Expression of the transcriptional fusions sirA-cat (A) and csrA-cat (B) was determined in the WT S. Typhimurium strain containing the vector pMPM-K3, or the plasmid pK3-SsrB, which expresses SsrB from a constitutive promoter. The CAT-specific activity was determined from bacterial cultures grown for 4 and 9 h in LB at 37°C. Data represents the mean with standard deviation of three independent experiments. EMSAs were performed to examine whether SsrB binds to the DNA fragments in the sirA-cat (C) and csrA-cat (D) fusions. The DNA fragments were incubated with increasing concentrations of purified 6H-SsrBc (0, 0.5, 1, 1.5 and 2 σM). DNA-protein complexes are indicated by an asterisk. (TIFF) [file ppat.1006497.s001.tiff]

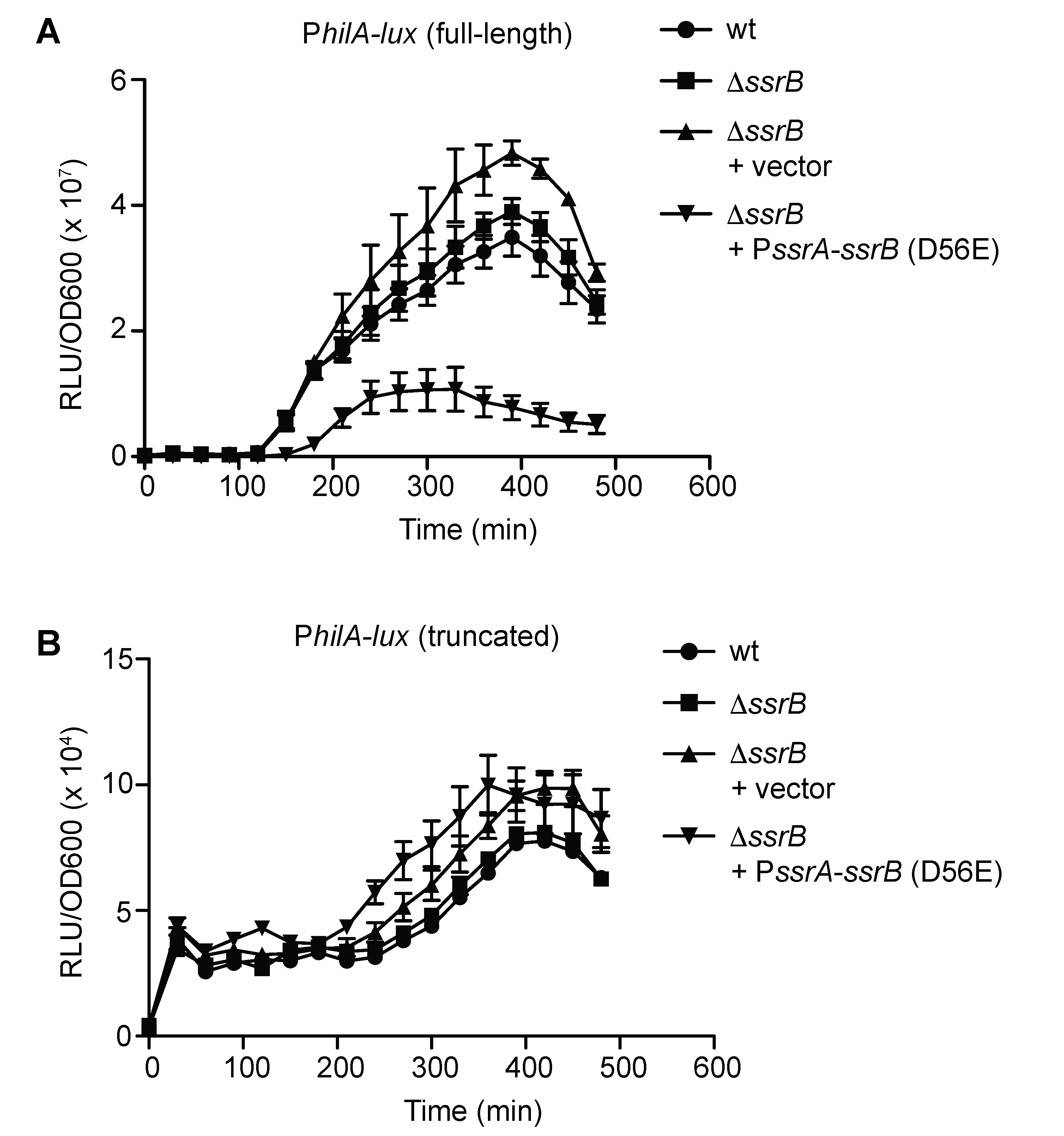

Supplement: S2 Fig — Expression of the hilA-lux-740+350 (full length) (A) and hilA-lux-36+446 (truncated) (B) transcriptional fusions was determined in the WT S. Typhimurium strain and its isogenic ΔssrB mutant containing or not the pWSK129 vector, or the pPssrA-ssrB (D56E) plasmid expressing SsrB with the D56E mutation, from its native promoter that is located upstream of ssrA. Luminescence (RLU) was quantified from bacterial cultures grown in LB at 37°C. RLUs were normalized to OD600 at each time point. Data represents the mean with standard deviation of three and two independent experiments for (A) and (B), respectively. (TIFF) [file ppat.1006497.s002.tiff]

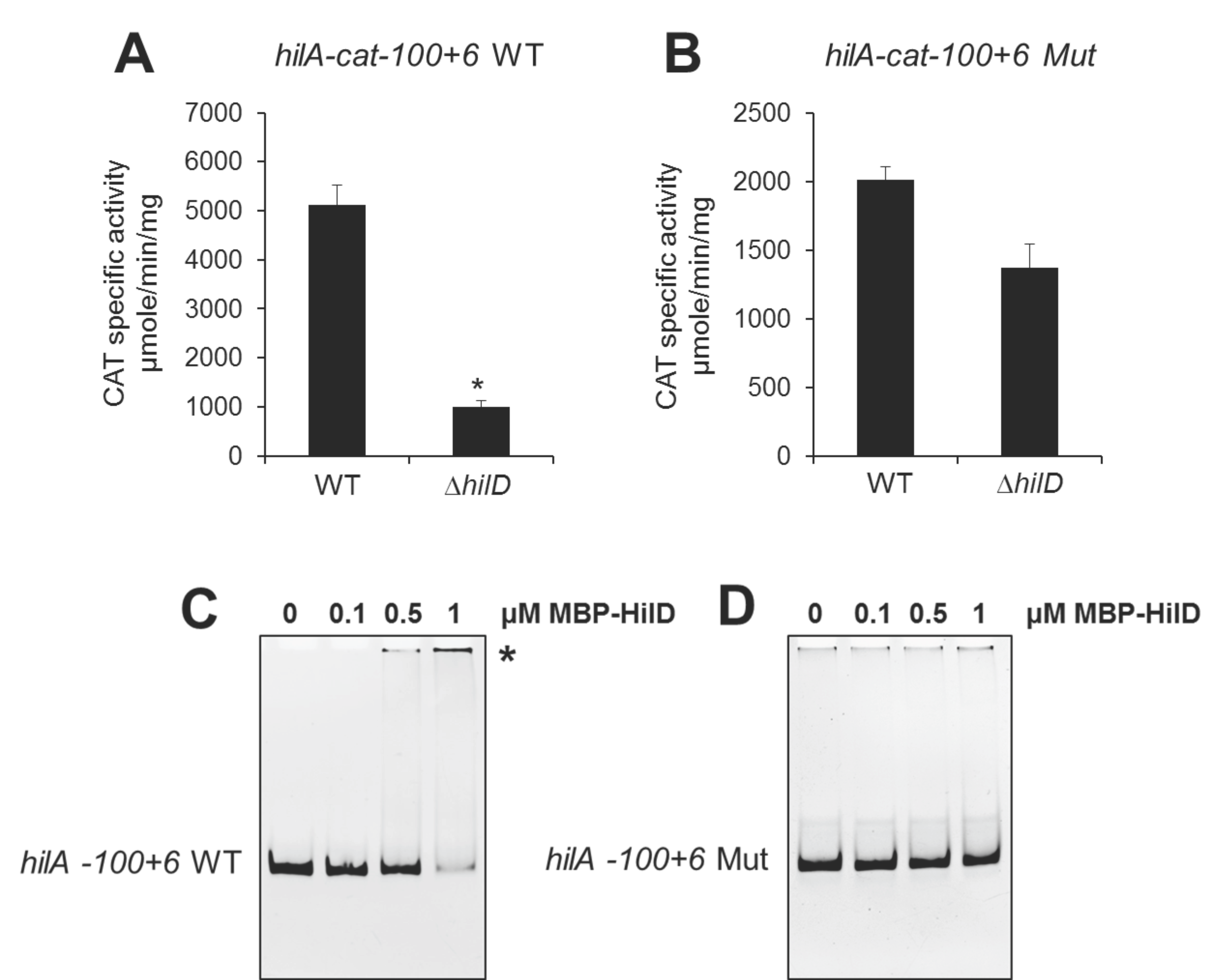

Supplement: S3 Fig — Expression of the hilA-cat-100+6 WT (WT SsrB binding site) (A) and hilA-cat-100+6 Mut (mutated SsrB binding site) (B) fusions was determined in the WT S. Typhimurium strain and its isogenic ΔhilD mutant. The CAT-specific activity was determined from bacterial cultures grown for 9 h in LB at 37°C. Data represents the mean with standard deviation of three independent experiments. *Statistically different values relative to the WT strain, P < 0.0005. EMSAs were performed to analyze the interaction of HilD with the hilA DNA fragments carried by the hilA-cat-100+6 WT (C) and hilA-cat-100+6 Mut (D) fusions. The DNA fragments were incubated with increasing concentrations of purified MBP-HilD (0, 0.1, 0.5 and 1 μM). DNA-protein complexes are indicated by an asterisk. (TIFF) [file ppat.1006497.s003.tiff]

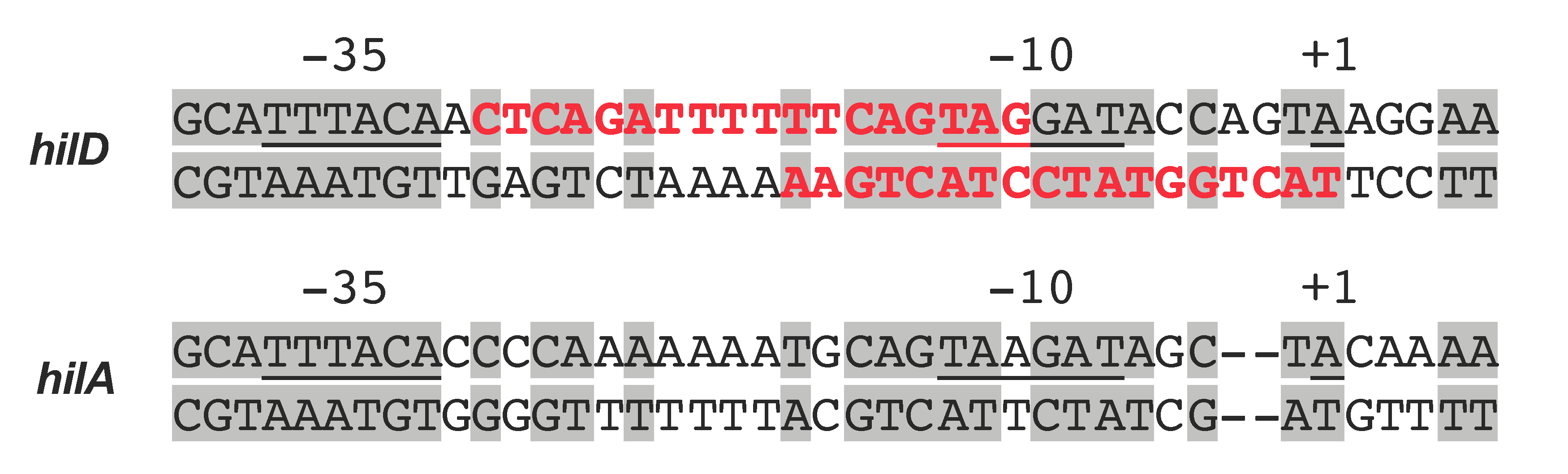

Supplement: S4 Fig — Common nucleotides are indicated by shading. The two predicted SsrB binding sites in hilD are shown by red letters. The transcriptional start site (+1) and the -35 and -10 promoter sequences are underlined. (TIFF) [file ppat.1006497.s004.tiff]

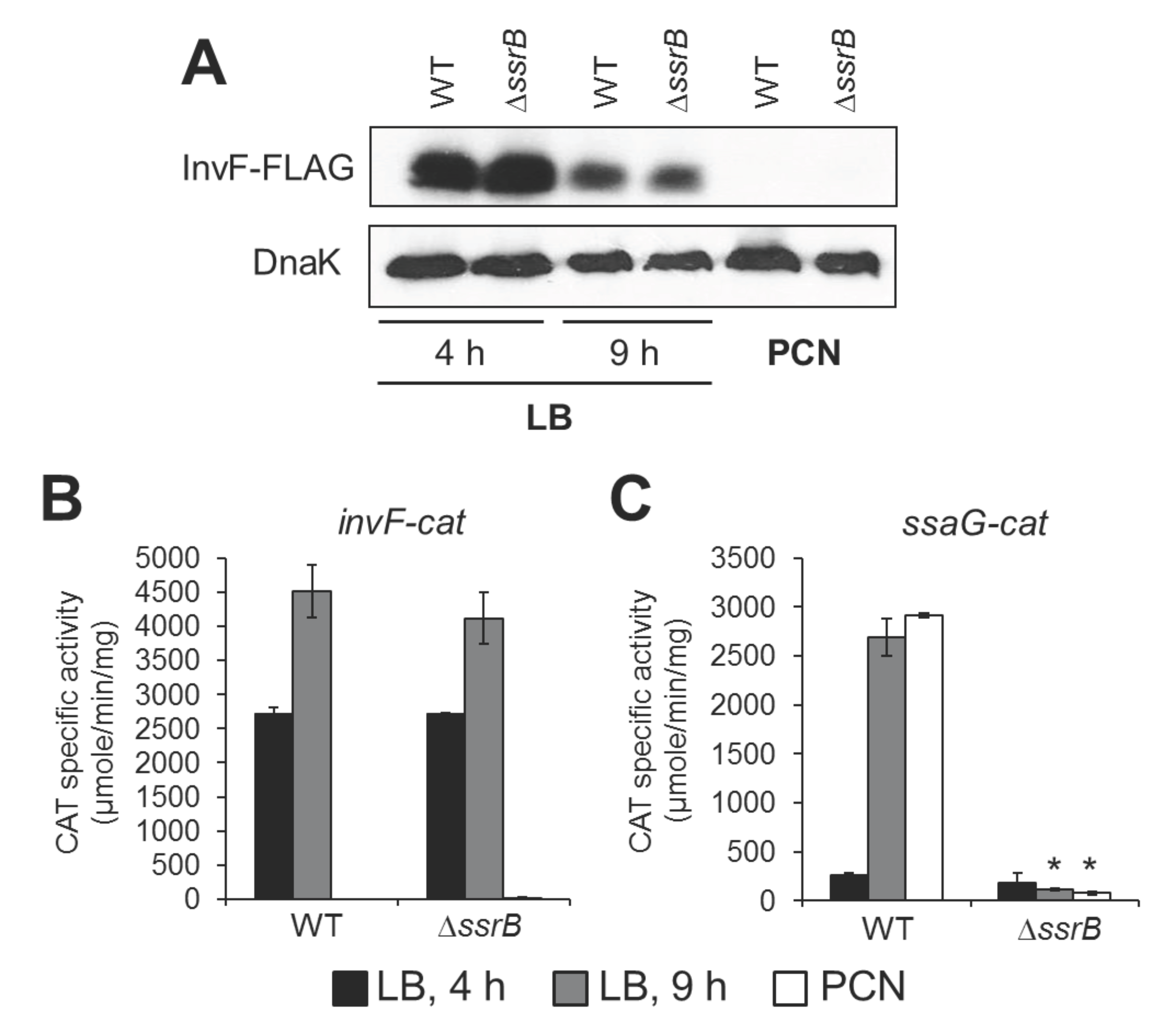

Supplement: S5 Fig — (A) The expression of InvF-FLAG was analyzed by Western blot in the WT S. Typhimurium strain and in a ΔssrB mutant, using monoclonal anti-FLAG antibodies. As a loading control, the expression of DnaK was also determined using monoclonal anti-DnaK antibodies. Expression of the invF-cat (B) and ssaG-cat (C) transcriptional fusions was measured in the WT and ΔssrB strains with chromosomally FLAG-tagged invF. Data represents the mean with standard deviation of three independent experiments. *Statistically different values with respect to the WT strain are indicated, P < 0.0005. Expression of InvF-FLAG, and the invF-cat and ssaG-cat fusions was determined from bacterial cultures grown for 4 and 9 h in LB or at OD600 of 0.3 in PCN, at 37°C. (TIFF) [file ppat.1006497.s005.tiff]

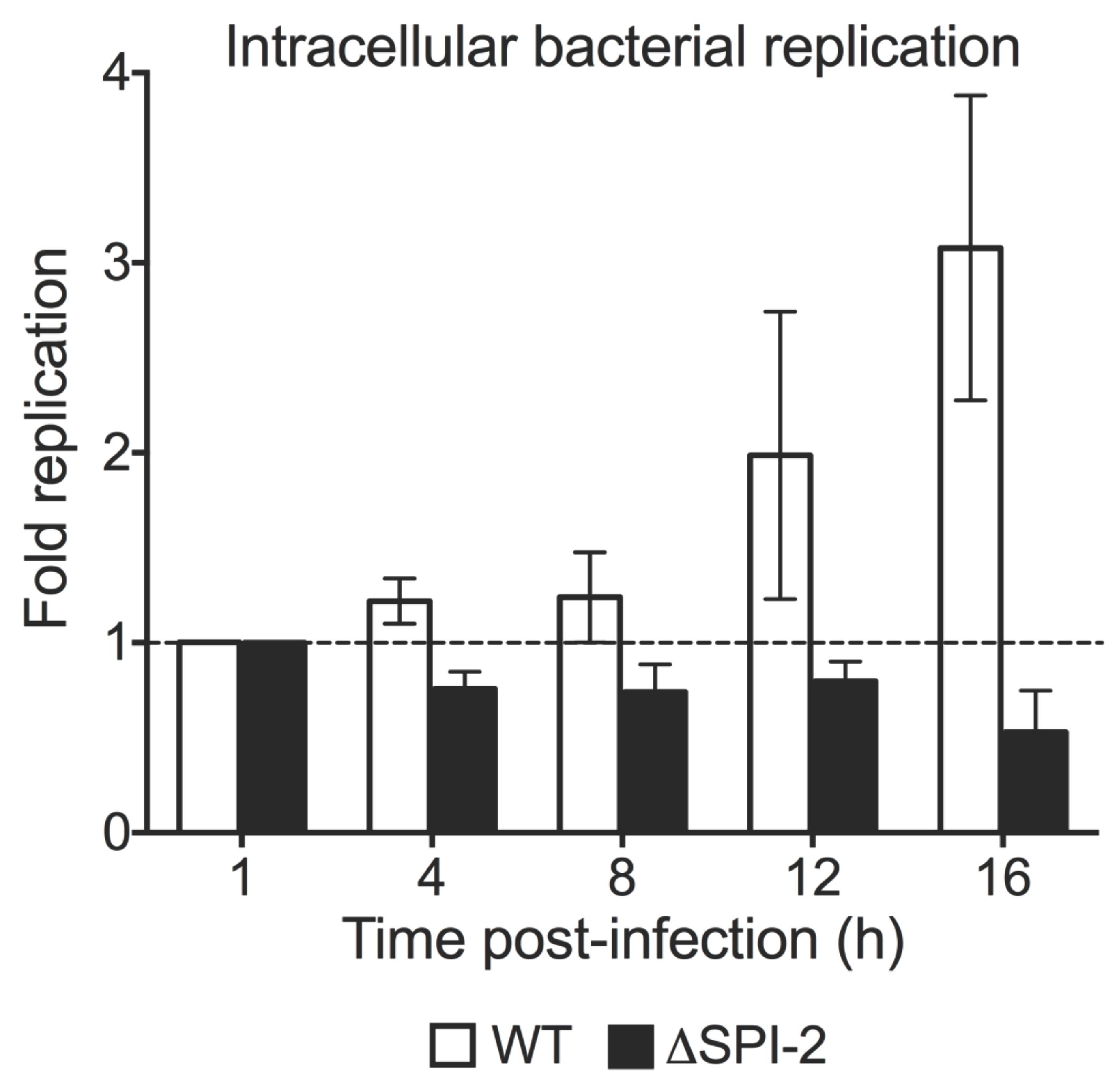

Supplement: S6 Fig — Fold-replication represents the CFUs recovered at the different post-infection times relative to the CFUs at 1 h post-infection for each strain. The dashed line is used to distinguish between increased and decreased replication levels. Data represents the mean with standard deviation of three independent experiments. (TIFF) [file ppat.1006497.s006.tiff]

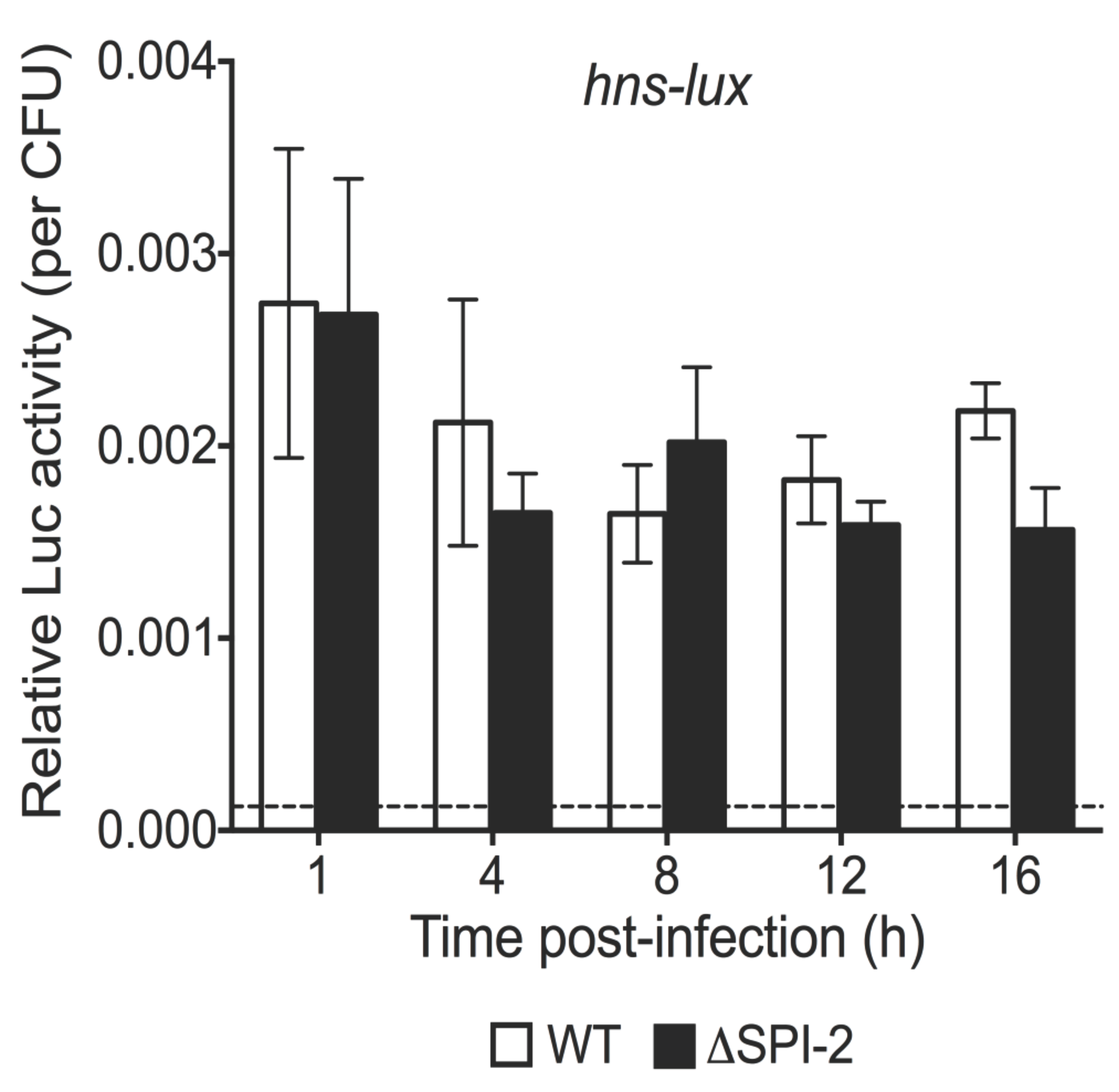

Supplement: S7 Fig — The intracellular expression of the hns-lux transcriptional fusion was examined in the WT S. Typhimurium strain and its derivative ΔSPI-2 mutant (lacking SsrB) in RAW264.7 murine macrophage-like cells. Luminescence was quantified and normalized to CFU counts at 1, 4, 8, 12, and 16 h post-infection. The dashed line represents the relative luminescence per CFU of the WT S. Typhimurium strain with the promoterless pCS26-Pac vector in RAW264.7 cells. Data represents the mean with standard deviation of three independent experiments. (TIFF) [file ppat.1006497.s007.tiff]

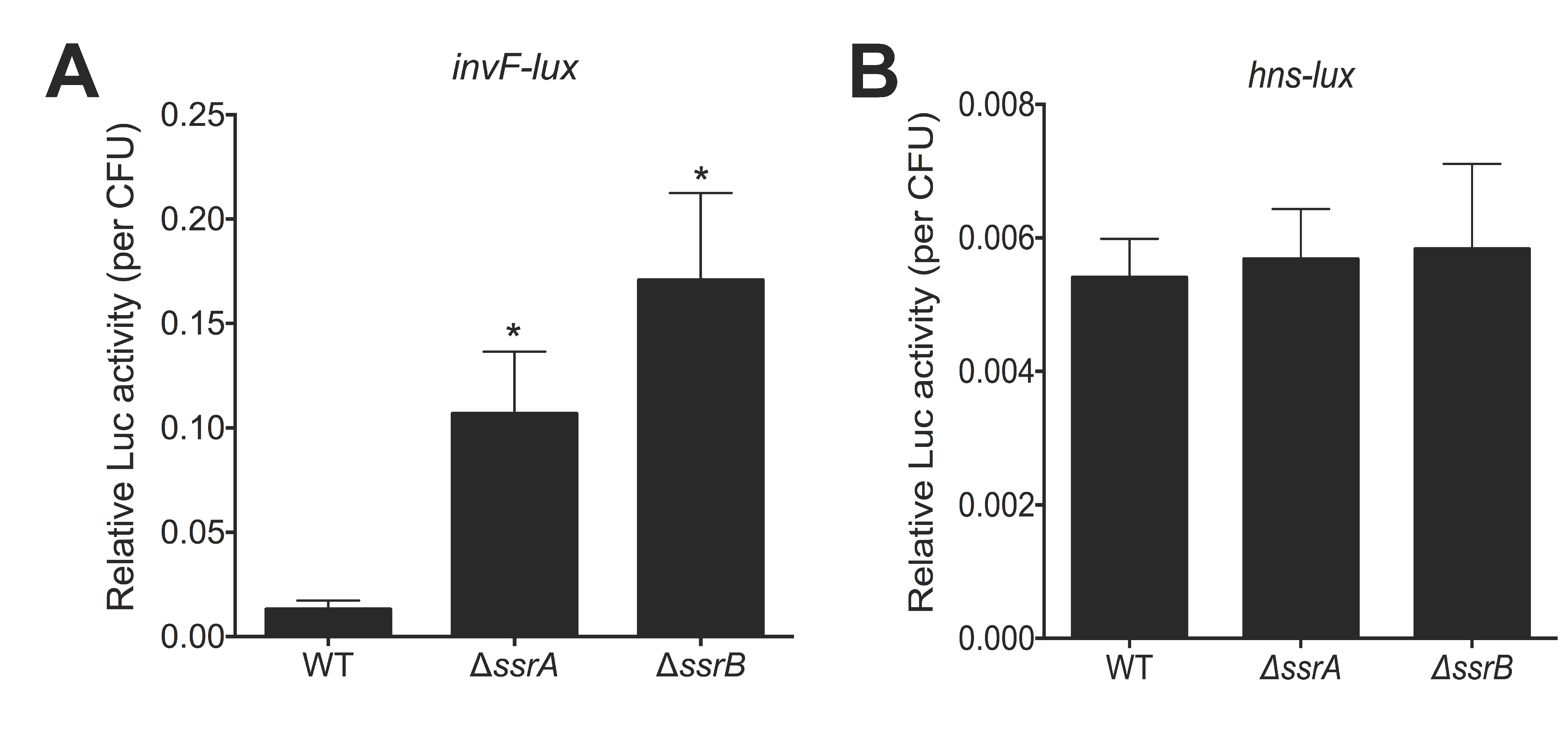

Supplement: S8 Fig — Expression of the invF-lux (A) and hns-lux (B) transcriptional fusion was analyzed in the WT S. Typhimurium strain and its isogenic ΔssrA and ΔssrB mutants inside RAW264.7 murine macrophage-like cells. Monolayers of macrophages were infected with an equal number of bacteria of the respective Salmonella strain. At 16 h post-infection the cells were lysed and luminescence and CFU counts were determined as described in Materials and Methods. Data represents the mean with standard deviation of three independent experiments. *Statistically different values with respect to the WT strain, P < 0.005. (TIFF) [file ppat.1006497.s008.tiff]
